# Attitudes and preferences towards screening for dementia: a systematic review of the literature

**DOI:** 10.1186/s12877-015-0064-6

**Published:** 2015-06-16

**Authors:** Steven Martin, Sarah Kelly, Ayesha Khan, Sarah Cullum, Tom Dening, Greta Rait, Chris Fox, Cornelius Katona, Theodore Cosco, Carol Brayne, Louise Lafortune

**Affiliations:** Cambridge Institute of Public Health, University of Cambridge, Cambridge, UK; Academic Unit of Psychiatry, University of Bristol, Bristol, UK; Institute of Mental Health, University of Nottingham, Nottingham, UK; Research Department of Primary Care and Population Health, UCL Medical School, UCL, London, UK; Faculty of Medicine and Health Sciences, Norwich, UK Medical School, University of East Anglia, Norwich, UK; Division of Psychiatry, University College London, London, UK

**Keywords:** Patient and public involvement, Screening, Dementia, Attitudes, Preferences, Oldest-old

## Abstract

**Background:**

Population screening might provide a mechanism to enable early detection of dementia. Yet the potential benefits, harms or acceptability of such a large-scale intervention are not well understood. This research aims to examine the attitudes and preferences of the general public, health care professionals, people with dementia and their carers towards population screening for dementia.

**Methods:**

A systematic review of the international literature was undertaken. A search of fifteen bibliographic databases was conducted (up to 12 July 2012; no language restriction) using terms related to dementia, screening, specific screening tools, case finding, and attitudes and preferences; genetic screening and biomarkers were excluded. All study designs were included except opinion-based papers. Included papers were doubly quality assessed and thematically analysed using NVivo.

**Results:**

29,910 papers were identified of which 29 met the inclusion criteria. We identified seventeen themes relating to the 3 phases of the screening process (pre-, in- and post-screen) – none emerged as more of a facilitator than a barrier to the acceptance of dementia screening. Seven themes emerged in relation to the patient, carer and general population: existing health state; lifestyle and life view; awareness of dementia; role of clinician; communication; benefit; and role of the family. Ten themes emerged in relation to the clinician and healthcare professional: patient’s existing health and comorbidities; awareness of dementia; confidence; duration of patient contact; suitability of screening tool; cost; disclosure; time; treatment and prognosis; and stigma.

**Conclusions:**

As for all screening programmes, screening for dementia raises complex issues around preference and choice for clinicians and the public, and it is unclear what specific factors promote or reduce screening acceptance the most. Overall, the level of evidence is low, few large scale studies have been undertaken and none were conducted in representative samples, all affecting the generalizability of identified themes across healthcare contexts. Nevertheless, our findings suggest that population screening for dementia may not be acceptable to either the general public or health care professionals, and highlight where focused efforts are needed to gain insights into dementia specific issues.

**Electronic supplementary material:**

The online version of this article (doi:10.1186/s12877-015-0064-6) contains supplementary material, which is available to authorized users.

## Background

Dementia is a condition of chief importance to ageing societies in terms of impact and cost, and it’s timely detection a clinical, research, and political priority. In a recent UK Department of Health report [1] it was estimated that 45 % of patients who might meet the criteria for dementia in any given population still do not receive a formal diagnosis or receive it too late to be clinically useful. Systematic population-level screening (a process of identifying a condition amongst a population of apparently healthy individuals) has been suggested as a possible mechanism to enable early detection of dementia in people who may be at increased risk [2–5]. Currently guidance produced by the UK National Screening Committee [6], the Royal Australian College of General Practitioners [7] and the US Preventative Services Task Force [8] advises against the adoption of screening for the early detection of dementia and cognitive impairment because there isn’t enough evidence to ensure the overall benefits of the screening journey would outweigh the harms [6, 9, 10].

We conducted a review of the literature to systematically address one specific screening criterion proposed by Wilson and Jungner [10] (Additional file 1) and the UK National Screening Committee [6]; “that the test should be acceptable to the population”. We broadened the concept of acceptability to include perceived risks and benefits arising from population screening, as well as attitudes, preferences and values of patients, carers, the general public and health care practitioners. Appraising acceptability broadly is important because it can reveal critical equity, ethical and social issues relevant to the development of new screening programmes, and highlight how informed choice, confidentiality and respect for autonomy might be ensured. Furthermore, these views are likely to impact on decision-making processes, compliance with the programme and ultimately the expectations placed upon a screening programme in terms of effectiveness and cost effectiveness. The current systematic review of the scientific literature is the first to evaluate the attitudes and preferences of the general public and health care professionals regarding population screening for dementia.

## Methods

To ensure transparent reporting on how we selected and analysed the literature, this review meets PRISMA (Preferred Reporting Items for Systematic Reviews and Meta-Analyses; http://www.prisma-statement.org) guidelines [11] (Additional file 2) and was conducted according to a pre-defined protocol (Additional file 3).

### Search strategy

The search strategy was developed with input from clinicians, health scientists and an information specialist. Electronic searches of bibliographic databases conducted on 16th July 2012 include: (1) terms relating to dementia; (2) terms relating to screening and case finding; and (3) terms related to attitudes and preferences. The search strategies covered all types of dementia, had no time or language limits, and were applied to the following databases: MEDLINE, EMBASE, CINAHL, CDR, Cochrane and Campbell Collaboration databases; PsycINFO, Social Sciences Citation Index, Web of Science, Bibliomap, DoPHER, TRoPHI. Reference lists of all included studies were scanned for relevant articles. The Medline search strategies for patients, carers and health care practitioners are presented in Additional files 4 and 5.

### Inclusion and exclusion criteria

Studies where the primary or secondary objectives were to explore, describe or explain the attitudes and preferences to screening for dementia (from the point of view of health and social care professionals, members of the general public, people with dementia, informal or formal carers) were included irrespective of their age, education, cognitive or dementia diagnostic status, or the setting in which the study was conducted. In addition, studies that assess health professionals’ perceptions, views and experiences were also included.

All study designs (qualitative, quantitative randomized experimental, quantitative non-randomized controlled, quantitative observation, and mixed methods) were eligible for inclusion. Opinion-based papers were excluded. Only published sources were considered.

Not all screening tests were considered for inclusion. This review only includes pen and pencil tests or any tests that can currently be easily administered in primary or community care settings to screen for dementia. We did not consider screening procedures that involve genetic tests or biomarkers. This is because such tests are being researched as potential pre-dementia “diagnostic” tests or as post-dementia diagnostic subtyping tests. Therefore they are not at present suitable for use as screening tests for the dementia syndrome at the population level. We also excluded screening to detect persons with MCI (Mild Cognitive Impairment) who do not meet the criteria of dementia, because MCI is variably defined and there is a lack of clarity around diagnosis of this condition [12].

### Outcomes

Primary outcomes - Perceptions, views and/or attitudes and/or experiences of patients and carers, and health and social care professionals were analysed with particular attention given to 1) their experience of screening (receiving or administering), 2) their view of population screening as an intervention (positive, negative), 3) quotes in support of views and perspectives, and 4) outcomes (views considered by authors as being associated with positive or false negative results).

Secondary outcomes - Ethical, moral and cultural issues; practical implications in terms of knowledge, organisation of health and social care (e.g. accessibility of diagnosis services, information and support; etc.), resources and funding in the context of the perception of patients, carers and practitioners.

### Identification of studies

All the citations identified in the search were downloaded into EndNote and screened for inclusion by two reviewers, who worked independently. All titles and abstracts were screened for inclusion by Steven Martin and Louise LaFortune. The full text of articles identified as either relevant or possibly relevant from the title and abstract were obtained and assessed to determine whether it met the inclusion criteria. Native speakers assessed non-English papers. Discrepancies between the authors were resolved via discussion at both stages. As screening for dementia may be part of a diagnostic process a number of relevant papers related to diagnosis of dementia were included up to full paper stage to examine if any references to population screening were made.

### Quality assessment

Two reviewers independently assessed study quality using a checklist adapted by Bunn et al. [13], building on the Spencer et al. [14] framework for assessing quality; the overall reliability and usefulness of the study to the research questions was graded as low, medium or high. We included all studies regardless of their quality. As a broad range of study designs have been used in this area of healthcare, the use of a single checklist, in contrast to individual checklists for each study design, was considered more appropriate.

### Data extraction

A data extraction form was developed and tested by two authors (SM, LL). Data were then extracted by two authors (SM and SK). A summary of data extracted can be found in Tables 1 and 2, full data extraction is located online in supplementary material (additional file 6).

**Table 1:** Summary table of included studies

First Author	Country	N.	Population	Study type	Quality rating
Boise L (2010)	USA	199	Clinicians and medical assistants	Intervention	Medium
Boise L (1999)	USA	78	Primary care physicians	Focus groups	High
Bond J (2010)	EU	Not reported	Clinicians, payers and general public	Survey	High
Borson SJ (2007)	USA	26	Medical assistants	Intervention	High
Boustani M (2011)	USA	206	Caregivers and non-caregivers	Survey	High
Boustani M (2008)	USA	315	Primary care patients’	Survey	High
Boustani M (2003)	USA	318	Older adults	Survey	High
Brodaty H (1994)	Australia	1473	General practitioners	Survey	Medium
Bush C (1997)	USA	360	Primary care physicians	Survey	Medium
Cahill S (2008)	Ireland	307	General practitioners	Survey	Medium
Carpenter CR (2011)	USA	55	Physicians and Nurses	Survey	Medium
Dale W (2006)	USA	149	Adults	Survey	Medium
Dale W (2008)	USA	199	Older adults	Intervention	Medium
Downs M (2000)	UK	278	General practitioners	Survey	High
Fowler R (2012)	USA	554	Primary care patients’	Survey	High
Galvin JE (2011)	USA	1024	Health care professionals	Pre-post test	High
Galvin JE (2008)	USA	1039	Older adults	Survey	Medium
Hansen EC (2008)	Australia	24	General practitioners	Focus groups	Medium
Holsinger T (2011)	USA	345	Primary care patients’	Survey	High
Iliffe S (1994)	UK	412	Older adults	Survey	Medium
Iliffe S (2003)	UK	247	Health care professionals	Workshop and survey	Medium
Iracleous P (2010)	Canada	249	Primary care physicians	Survey	Medium
Justiss MD (2009)	USA and UK	245	Older adults	Survey	High
Krohne K (2011)	Norway	18	Older adults	Observational	Medium
Lawrence JM (2003)	USA	787	Clinicians and community-dwelling individuals	Intervention and survey	Medium
Manthorpe J (2003)	UK	Not reported	Health care professionals	Workshop and survey	Medium
Martinez-Lage P (2010)	EU	500	Physicians	Survey	Medium
Welkenhuysen M (1997)	Belgium	167	Medical students	Survey	Medium
Williams CL (2010)	USA	119	Adults	Interview	Medium

**Table 2:** Summary of themes in included studies

Theme	Reference
**Pre-screen**	
Stigma and awareness of disease	22, 24, 27, 29, 30, 31, 33, 34, 35, 36, 38, 39, 43, 43, 47, 50
Role of family	27, 31, 32, 34, 36, 37, 38, 39
Existing health	22, 27, 31, 34, 36, 40
Health insurance/financial/Employment/driving	21, 28, 35, 43
Duration of contact	46
Locality	37, 46
Current practice/practicalities	36, 38, 40, 42, 44, 45, 49, 50
Lifestyle and life view	21, 24, 26, 27, 28, 29, 33, 37, 48, 50
Training	22, 31, 34, 36, 39, 47, 49, 50
**In-screen**	
Time constraints	36, 41, 44, 45, 46
Inaccuracy of test	41, 45
Cost	38, 45
Communication	22
**Post-screen**	
Lack of change in prognosis, treatment and patient benefit	21, 23, 24, 26, 27, 28, 29, 31, 32, 33, 35, 36, 37, 38, 39, 41, 42, 44, 45, 47, 48, 49
Role of support	30, 37

### Data analysis

Two approaches to analysis were adopted. A thematic approach [15] was used to analyse people’s (patients’, carers’, the general public’s and health professionals’) attitudes, behaviours, value systems, concerns and perceptions with regard to screening for dementia. Questionnaire and survey data were extracted and summarised. Source data management and thematic analysis was carried out within the computer-assisted qualitative data analysis software, NVivo (version 9.2) and Excel. Two researchers independently coded the data (SM and SK). The data was coded into topic areas and then further analysed and developed into themes. Where there were differences between the researchers, themes were further discussed and agreed. Due to the predominantly qualitative nature of the data, and the presence of clinical, methodological and statistical heterogeneity, it was not appropriate to conduct a meta-analysis. This approach supports our purpose because it involves discussions to establish consensus on the format of data extraction and extraction of data around key themes. A further advantage is the opportunity it provides to revisit the context of the quotation within a study very easily. This means that the exact content of the discussions are examined more closely and different aspects of a ‘dense’ paragraph of data can be assigned to several themes.

A narrative synthesis of data from all included studies was undertaken by SM. This was done to provide a detailed summary and comparison of attitudes and preferences across studies. We then analysed the findings and discussion sections of the papers by identifying key themes represented in data (i.e. quotations) and the statements made within the discussion. We did not adopt a line-by-line analysis in which codes are assigned to each line of text as we did not feel that such intense scrutiny of content would have enhanced our collection of descriptive and interpretive data. To deal with the apparent subjectivity of the process, the themes identified by both reviewers were compared and discrepancies were discussed. This approach to data extraction increases the likelihood of achieving data saturation [16, 17].

Data were divided into two broad categories a) themes impacting on the attitudes and preferences of patients, carers and the general public, and b) themes impacting on the attitudes and preferences of clinicians and healthcare professionals. Data was organised in this way because the available evidence derives from these populations. There was therefore no need to pre-conceive groupings.

We paid particular attention to i) whether someone had been screened for dementia or had a diagnostic assessment for dementia which could have influenced their perceptions of the potential harms and benefits of screening, and ii) whether the type of tests and/or setting influences perceptions of physicians with respect to the acceptability, potential harms and benefits of screening for dementia; iii) the year the study was published to detect potential trends over time.

## Results

### Search results

**Fig. 1:**
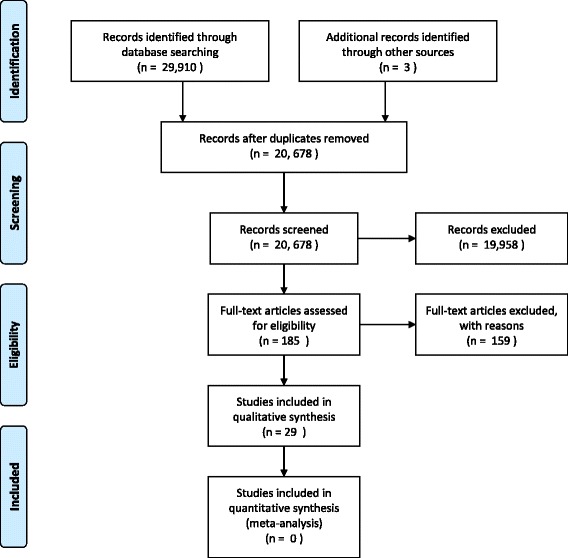
Flow of information through different phases of the systematic review

The database searches generated 20,678 titles (Fig. 1), which were then screened for inclusion. Given the broad use of the terms screening and diagnostic, we carefully assessed 720 abstracts and the full text of 185 papers. In addition, three additional papers were identified from hand and grey literature searches. We included 29 studies looking at attitudes and/or preferences towards screening for dementia. The reasons for exclusion, at full paper, were: not about screening (n = 146), including 15 reviews that did not examine dementia screening, not primary research (n = 11); unobtainable (n = 2). Three non-English language papers were translated but excluded at full paper sift [18–20].

### Sample characteristics

Nine papers involved patients or people with dementia in their research. A total of 2,575 patients or people with dementia were included in this review. Two studies involved a total of 331 carers in their research. Study sizes ranged from 81 to 250. A number of studies reported unclear sample sizes. There were no studies based on samples representative of the general population; however, five studies were based in the community and had participants recruited from the general public. In total 1,977 people from the general public were included in this review (study sizes ranged from 125 to 1,039 people).

Health care professionals constituted the largest group of participants in the identified studies. Fifteen studies included a total of 5,132 clinicians or health care practitioners. One paper did not provide a clear description of the study sample. Number of participants ranged from four to 1,473. General practice, including both general practitioners and practice nurses was the most frequent research setting (11 studies). Settings for the remaining studies with healthcare professionals included: community nursing, geriatric specialists, community mental health, emergency department, outpatient clinic and university – one in each.

### Study characteristics

Studies took place in Australia (n = 3), Belgium (n = 1), Canada (n = 1), Ireland (n = 1), Norway (n = 1), UK (n = 5) and USA (n = 15). One study had both UK and USA sites and two studies were based across five European countries (France, Germany, Italy, Spain and the UK). The oldest included study was published in 1994, the most recent published in 2012. In most cases the ‘test’ was hypothetical, or described in very general terms (i.e. a screening test). No studies were included which examined the attitudes and preferences, or acceptability of a specific existing tool. There were a range of quality ratings; 18 studies were rated as medium quality and 11 were rated as high (Table 1). Full quality assessment scores are presented online in the supplementary evidence (Additional file 7).

### Risk of bias

Due to the heterogeneity of included studies the pooling of results is inappropriate for this review. Given that qualitative data were extracted no test to assess homogeneity (e.g. chi-squared test for homogeneity, I^2^, random effects model, Egger regression test, Hedges-Olken) is appropriate. Sources of financial support for included studies can be found on line in supplementary material (Additional file 7).

### Attitudes and preferences

**Fig. 2:**
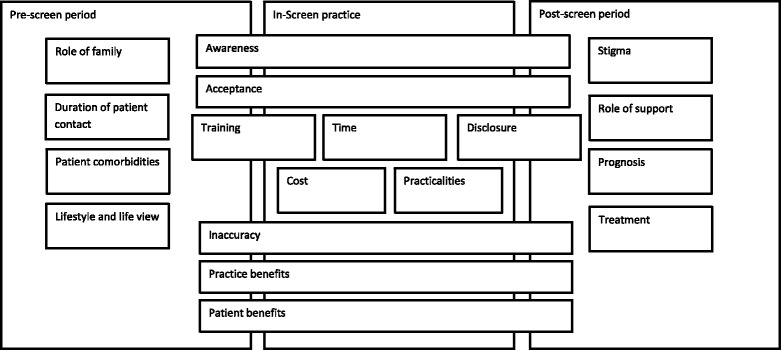
1) Pre-screen, in-screen and post-screen refers to three stages of the screening process. 2) Each box represents themes that emerged repeatedly from analysis for public/carers and health care professionals

Three stages of the screening process were identified: a) the pre-screen period, b) the in-screen, and c) the post-screen period. At each stage of the screening process a number of factors impact on an individuals’ decision to screen or be screened, and are relevant to the acceptability of population-level screening for dementia (Fig. 2). Seven key themes emerged from studies with the patient, carer and general population: 1) existing health state; 2) lifestyle and life view; 3) awareness of dementia; 4) role of clinician; 5) communication; 6) benefit. Ten key themes emerged in relation to the clinician and healthcare professional: 1) patient’s existing health and comorbidities; 2) awareness of dementia; 3) acceptability; 4) duration of patient contact; 5) screening tool; 6) cost; 7) disclosure; 8) time; 9) treatment and prognosis; 10) stigma. These themes emerged repeatedly in the peer-reviewed literature and are discussed below.

#### Themes in relation to the patient, carer and general population:

##### Existing health state

Survey data [21] found that being healthier (as indicated by taking fewer than three medications) was associated with less willingness to accept dementia screening and that having some type of cognitive difficulty was associated with more willingness to be screened for dementia. A qualitative study [22] also showed that a person’s existing health state and perceived susceptibility to illness may impact on the acceptability of screening for dementia and on ability to undergo screening tests, as exemplified in the following quote:

*“It wasn’t that it couldn’t be done, but at my age… I got tired – this is a weariness I carry with me everywhere (and it kicks in every time) I’m exposed to something complicated.”* [22]

##### Lifestyle and life view

The data on lifestyle and views of patients are predominantly derived from survey responses. Surveys consisted of validated scales and items designed by the research teams. No studies contained substantial qualitative components. While some data [23–27] suggest that some respondents have no concerns and are pleased to have their memory evaluated, other results [21] suggest that few people would agree to routine screening for memory problems for reasons such as stigma [28–30]. It is important to take note that no patient or carer mentioned the word stigma; this word was adopted by researchers in their attitudinal scales.

##### Awareness of dementia

There are issues around awareness and public education [31]. The quotations below illustrate that some individuals have a low level of awareness regarding the screening test. In these cases there have been some misunderstandings around both the reasons for a screen and the implications of test results [22, 31, 32]. While we are unable to say what proportion of individuals have not fully understood or poorly interpreted the screening test, it is the responsibility of health care professionals to raise the issue and ensure consent is given.

*“It [the screening test] is probably to do a little bit of research on what we remember, and… if our heads are where they are supposed to be…”* [22]

*“…she tested my head, that’s what she did.”* [22]

*“I got the impression that I passed the test. Yes. Or you could say it was examination questions, right?”* [22]

##### Role of clinician

This theme emerged from two studies, and as the quote below shows, a person’s acceptance of dementia may be influenced by the role of the clinician [32]; *“It’s okay to be screened’cause then [the occupational therapist] gets to see what I really need help to do”* [22]. A clinician needs to engage with the individual to outline what their role is, how they will conduct the test and what the outcome may be, essential preparatory work in order to manage patient experience and expectations.

##### Communication

In one study [22] a number of patients reported uncertainties around the test and some of these individuals were unable to recollect the screening was explained to them beforehand, or how the results were presented afterwards. While we cannot substantiate that poor communication on behalf of clinicians is responsible for levels of confusion, the patients’ were still reliant on their own interpretation of the screening test, its purpose and the potential outcome. The patients also reported screening to be strenuous or stressful, mostly due to a perceived pressure to perform well on the test.

*“No, I wasn’t told. I don’t know.”* [22]

*“[I]f I was to guess (…) it has something to do with memory?”* [22]

##### Benefit

Where data is available [22, 26, 28, 30, 33, 34] it seemed that caregivers and the general public believe there are a number of benefits to screening for dementia, including treatment and financial benefits. However, in one study [35] nearly half of the patients who screened positive for cognitive impairment refused a diagnostic evaluation.

*“I’m over 50 with no children. I need to know how to be prepared.”* [34]

*“[they can] catch it before it’s too late.”* [34]

Studies that reported the ability of families to plan and make arrangements are often cited but, little qualitative evidence was found to support this claim. Most evidence is derived from questionnaire responses.

##### Role of family

There was good evidence to suggest that the family plays an important role in decisions whether or not to undergo screening [24, 31, 32, 34, 36–39]. The influence of the family is dynamic and can include influencing decisions to consult health care professionals in primary or secondary care. The family may also act as a prompt, recognising that an individual may have issues related to the onset of the disease. The quote below is from the perspective of a primary care physician, discussing how important the family is to the patient when deciding whether or not to consult or undergo screening.

*“I’d say in 90 % of the cases it’s the family [that brings the dementia to my attention].”* [36]

#### Themes in relation to healthcare professionals:

##### Patient’s existing health and comorbidities

Four studies [30, 34, 36, 40] showed that in most cases the doctors felt that the individual was too ill to proceed with a full assessment or to use screening instruments. The clinicians tend to address other more easily treatable issues first rather than the dementia because they report making more impact.

*“When we do see people for dementia, it is common that they have ten other medical problems. There’s usually something else going on - dementia or memory problems is right at the bottom of the list, in terms of things to address.”* [34]

##### Awareness of dementia

Lack of awareness of dementia on the part of healthcare professionals was reported to be a barrier to recognising symptoms. Studies [36, 39] found that the primary barriers were symptom recognition, physician attitudes, and constraints in contemporary medical practice. It appears that whilst the presentation and recognition of symptoms do not impact on decisions to screen (individuals are asymptomatic at point of test) it is the attitudes, rather than knowledge, which may determine whether physicians conduct a full assessment.

##### Acceptability

Two studies [35, 41] found that acceptability levels for screening was high in staff members, conversely a number of studies [33, 39, 42, 43] asked clinicians and other HCPs and found that clinicians were undecided or negative when asked if cognitive screening in primary care would lead to better outcomes. In one study, female clinicians were more likely to have a positive attitude towards screening [44].

##### Duration of patient contact

Our findings suggest that clinicians may not feel confident about screening people with whom they have little or no relationship [36, 45]; conversely they also feel some apprehension towards screening patients with whom they have had a long relationship [34, 45]. The finer detail of why this may be the case was not presented in the literature, but there could be a number of possibilities including issues around lack of understanding of prognosis, poor treatment options, lack of transparent care pathways etc. The quotes below illustrate these points well:

*“I know I’m guilty of it, and suspect the rest of us are too . . . which is that, with someone who’s been your patient for a while and, you haven’t done a Mini-Mental State Exam on them…”* [45]

*“Very often you know these patients very, very well and have seen them over many years … and maybe you don’t notice, because of your lack of memory … their lack of memory and then it’s really only when a crisis occurs… something happens that sort of makes everybody stand back and say ‘Oh my God, it’s really obvious’ and made it difficult for most to administer the MMSE (Mini Mental State Examination).”* [45]

##### The screening tool

The lack of acceptable and accurate screening tools also provides a barrier to population screening for dementia for the general population [45–48]. In one study [48] both generalists and specialists reported that screening inaccuracy was the most important reason for not undertaking routine screening at age 65. When clinicians and health care professionals were asked about their preferences regarding tools, tests such as clock drawing [46] and the short Blessed [47] were identified.

*“The MMSE is quite distressing… to do with a patient you know, I think it’s quite an invasive test… I think part of the problem is that the minute you start doing it, it’s…very direct.”* [45]

##### Cost

There were a number of practicalities that made screening for dementia problematic, including the cost [38]. Cost concerns from clinicians were related to implementation, disruption to current working practices as well as costs of additional staff and/or infrastructure [38]. Costs concerns from patients included rising health and travel insurance and other benefits. Costs of direct care and financial implications of indirect care were also discussed.

##### Disclosure

Screening involves communicating the outcome to the patient so issues of diagnosis disclosure inevitably arise. The difficulties were perceived as closely connected with a clinician’s duration of contact with their patient, with particularly problematic implications around lack of prognosis, poor treatment options, a lack of transparent care pathways and the patient’s existing state of health [30, 43, 46]. Evidence suggests that clinicians believe interventions are “timely” when required in response to a patient’s functioning or cognition prompting them to present to medical attention, rather than an approach that encourages disclosure to all regardless of their existing needs [36, 37, 49]. Importantly, the identification of dementia was perceived as potentially harmful to some patients [37]. The following quotes illustrate the point:

*“I’ve walked out of the room lots of times going ‘I think something’s going on here but not pushed it, because what am I going to do? What am I going to tell the family?’ Well, they’re functioning okay in the home, I think they’ve probably got early dementia, but is it going to change anything? No. Can I do anything about it? No. So, why get everybody all excited when we’ll just keep a close eye on it.*” [36]

*“The family doesn’t want to hear, the patient doesn't want to hear. The ‘gradual decline of forgetfulness’ is a much better description to the patient and the family.”* [36]

##### Time

Lack of time to screen was a common theme that emerged in the literature [36, 41, 45]; however, in the single study [35] that examined the impact of a screening intervention on practice, none of the staff reported significant disruption to existing working practices.

##### Treatment and prognosis

Attitudinal barriers included the perception that nothing could be done for patients with dementia [34, 41, 49, 50] given the limited effectiveness of currently available treatments. It appears that for many clinicians, until there are effective treatments, there is little reason to assess patients for cognitive problems. A major finding [23] was the reluctance of clinicians to follow-up on a positive dementia screen. Clinicians often determined that the symptoms did not warrant a dementia work-up.

*“Sounds like there’s some message coming from somewhere [that doctors should be more] aggressive with early diagnosis… If that’s the case that needs to be communicated with some really good reasons. To offset the ‘I don’t want to know, the family doesn’t want to know…’ There [needs to be] something that changes the prognosis.”* [36]

##### Stigma

Some clinicians recognised the stigma associated with dementia and with Alzheimer’s Disease specifically. This finding may reflect the lack of effective available treatment options and the level of disease awareness in the population [30,36.38].

*“I have the most trouble discussing with the patients and families. I have no problem about cancer or other fatal diseases, but Alzheimer’s disease has a huge stigma associated with it.”* [36]

#### Differences between patients, public and clinician views

For patients, the key factors that impact on the acceptability of screening for dementia relate to the individual’s context such as their current health, lifestyle, life view and their knowledge of dementia. Where evidence does exist, patients have not been shown to consistently recollect either the screening test or the result, which raises ethical issues around consent and disclosure. Three themes – awareness, role of the clinician and communication – may potentially empower the patient to make informed choices which impact on their long-term health outcomes. For clinicians, a number of contextual issues (including current practice, poor prognosis, lack of treatment options, stigma and the duration of contact with the patient) and mechanisms (including the practicalities, awareness, confidence, accuracy of tools, cost, time, a patient’s existing state of health and issues around disclosure) appear to impact on acceptability and decisions to screen. Whilst the public are more concerned about what use a screening test will be to them immediately and in the short-term, clinicians have some paternalistic/protective concerns over the long-term impact of dementia screening. Awareness of dementia is important to patients, carers and health care practitioners.

## Discussion

### Summary of main results

This systematic review is the first to evaluate the attitudes and preferences of people with dementia, their carers, and the general public and health care professionals regarding population screening for dementia. We identified a relatively small number of papers despite using a broad search strategy.

At each stage of the screening process – before, during and after screening – a number of attitudinal factors impact on decisions about screening and the acceptability of population-level screening for dementia. However, not all these themes are expected to contribute equally in every situation since people make choices and take decisions according to their own values, experience and context.

### Strengths and limitations

The key strength of this review pertains to the extensive literature searches, entailing multiple search strategies for carers, patients and healthcare professionals and also including grey literature, which means we are confident that most of the published evidence has been captured. The analytic framework adopted in this review facilitated categories/themes to emerge from multiple sources of data and then an examination of the interrelations among these themes. There are, however, a number of weaknesses, related namely to the available evidence which impact on the generalizability of our findings.

Most studies have been undertaken in the USA and Canada, including some in the general population, however, results are mixed. Factors that appear to impact on the acceptability of a screening programme include stigma, misconceptions and poor levels of awareness around disease combined with the knowledge of poor prognosis and few treatment options; however, due to a relatively small number of papers of limited quality themes might be incomplete. Other factors included health insurance and other financial implications, and impacts on employment and driving. Evidence also suggests that a person’s lifestyle and attitude to health plays a role in decisions to screen. The perception and impact of stigma is a concern to patients, carers, the general public and health care professionals; however it is difficult to conclude from the literature identified to what extent the findings under individual themes (e.g. acceptability, disclosure) can be explained, at least in part, by underlying or unrecognised stigma. These findings may suggest that the stigma around Alzheimer’s disease and dementia requires tackling through education before a national screening programme could be developed and successfully implemented. Studies were examined to assess if there are any differences in the people’s attitudes toward dementia screening between the older studies and those more recent ones; however no differences were found. Economic costs are an important factor in determining the adoption of screening for dementia at policy level. This research team conducted a systematic review to assess the cost implications of screening for dementia - results will be published shortly.

No study was identified which assessed the attitudes and preferences of a sample representative of the UK general population. A number of small studies (n = 5) have been conducted in the UK which surveys the attitudes and preferences of health care professionals. These participants reported they saw small patient benefits through poor prognosis and few treatment options as impacting on the acceptability of a screening programme for dementia. Current practice, time constraints and practicalities of screening, as well as a patient’s existing health, also impact on decision-making. There are further issues around which tool is most appropriate to adopt. Current work, using a participant and public involvement strategy or adopting a qualitative research design, has examined more closely the findings of this review and contextualised them for a UK audience (soon to be published).

The level of evidence is quite low and few large-scale mixed-methods studies have been undertaken. In this review one medium quality study [19] provided substantial qualitative data on the attitudes and preferences of patients; and another high quality study [32] provided substantial qualitative data on the perspectives of health care practitioners. However, this brings a number of limitations including the contextual dependency and generalizability of the findings. While the attitudes and preferences reported in these studies may be credible and valid in the samples they recruited, their transferability to other populations remains unknown. While the review demonstrates that the acceptability of dementia screening found in different studies may vary, the magnitudes of these variations and their impact on screening decisions are difficult to determine. Findings may also be influenced by the prior views of researchers, though the dual structured process of paper selection, data extraction and analysis will have guarded against major biases. Future research may undertake assessments of equipoise of screening interventions for clinicians and study authors*.*

Although most of the clinicians and patients involved in the studies included in this review consider dementia a serious condition, many questioned the need to identify it early through population screening. Whilst the general public may have a more positive opinion of routine screening, this needs to be contextualised with other themes such as poor awareness and communication, and what is expected from the natural progression of the disease. Members of the general public may be more positive because they have a poor understanding of the disease, its natural progression and prognosis.

## Conclusions

The published literature on attitudes towards population screening for dementia is diverse and fragmented and it is difficult to draw clear conclusions from the data. Our review indicates that several factors may either enhance or limit the acceptability of screening for patients, carers, the general population, and clinicians. Attitudes and preferences are complex and multi-factorial and our findings suggest that population screening for dementia may be acceptable neither to the general public nor to health care professionals. Both groups express concerns about the means by which screening is performed, mutual trust, and uncertain outcomes. Policy makers should be cautious about the adoption of population screening for dementia without evidence and careful evaluation of benefits or risks, as noted by Le Couteur and colleagues [9].

This project highlights where focused efforts are needed to further our understanding of how to improve timely detection of dementia in the community. Given that individual variants appear largely responsible for decision-making, there needs to be a sensitive approach to the identification of dementia. This review focuses on any tests that can currently be easily administered in primary or community care settings to screen for dementia yet the public debate should encompass the attitudes and preferences of the general public and clinicians towards genetic screening for dementia (for which a parallel review is underway). We conclude by arguing that the challenge for health and social care professionals as well as policy makers is to engage with their patients and the public to understand in greater depth their expectations and perspectives on population screening for dementia.

## Additional files

Additional file 1:
**Wilson and Jungner classic screening criteria.**
Additional file 2:
**PRISMA checkist.**
Additional file 3:
**Systematic review protocol.**
Additional file 4
**Search strategy Medline Search – Patients & Carer Perceptions.**
Additional file 5:
**Medline Search – Practitioners Perceptions.**
Additional file 6
**Data extraction for included studies.**
Additional file 7:
**List of funders for primary studies.**

## Abbreviations

PRISMA: Preferred Reporting Items for Systematic Reviews and Meta-Analyses
MCI: Mild Cognitive Impairment
MMSE: *Mini Mental State Examination*
