# Supplementary material for: Attitudes and preferences towards screening for dementia: a systematic review of the literature
Source: BMC Geriatr. 2015 Jun 16;15:66. doi: 10.1186/s12877-015-0064-6 (PMC4469007; doi:10.1186/s12877-015-0064-6)
Supplement: Additional file 1: — Wilson and Jungner classic screening criteria. [file 12877_2015_64_MOESM1_ESM.docx]

**Appendix 1: Data extraction for included studies**

| **First Author  (Year)** | **Summary of results** | **Significance of outcome (statistical) based on data in paper** | **Significance of outcome (narrative) based on data in paper** | **Implications for policy, practice and future research based on authors conclusions** |
| --- | --- | --- | --- | --- |
| **Boise (2010).**  The rural older adult memory (ROAM) study: a practice-based intervention to improve dementia screening and diagnosis. *J Am Board Fam Med*. 2010;23(4):486-498. | Ninety-eight percent of respondents included in the sample reported no concerns or that they were pleased to have [their] memory evaluated.  Ninety-one percent responded that, in general, memory evaluation for older patients was a good idea  A major ﬁnding of this study was the reluctance of the clinicians to follow up on a positive dementia screen. Clinicians often determined that the symptoms did not warrant a dementia work-up. | **Practice-Level Data for Intervention Protocol** **Practice A** Patients >75 Years of Age Seen During Intervention Period (n) 156  Screen Forms Initiated (n [%]) 134 [86)] Patient Screens Completed (n [%]) 108 [81]  Positive Screens (n[%]) 68 [63] Patients Scheduled for Work-up (n [%]) 29 [43]  Patients Evaluated (n[%]) 28 [97] Patients Diagnosed with Dementia (n [%]) 7 [10] **Practice B** Patients >75 Years of Age Seen During Intervention Period (n) 152  Screen Forms Initiated (n [%]) 96 [63] Patient Screens Completed (n [%]) 82 [84]  Positive Screens (n[%]) 33 [40]  Patients Scheduled for Work-up (n [%]) 5 [15] Patients Evaluated (n[%]) 3 [60] Patients Diagnosed with Dementia (n [%]) 2 [6] **Practice C** Patients >75 Years of Age Seen During Intervention Period (n) 96  Screen Forms Initiated (n [%]) 87 [91] Patient Screens Completed (n [%]) 77 [88]  Positive Screens (n[%]) 40 [52] Patients Scheduled for Work-up (n [%]) 8 [20] Patients Evaluated (n[%]) 7 [88] Patients Diagnosed with Dementia (n [%]) 2 [5] **Practice D** Patients >75 Years of Age Seen During Intervention Period (n) 88  Screen Forms Initiated (n [%]) 83 [94] Patient Screens Completed (n [%]) 77 [93] Positive Screens (n[%]) 30 [39] Patients Scheduled for Work-up (n [%]) 15 [50]  Patients Evaluated (n[%]) 12 [80] Patients Diagnosed with Dementia (n [%]) 6 [20] **Practice E** Patients >75 Years of Age Seen During Intervention Period (n) 73 Screen Forms Initiated (n [%]) 50 [68] Patient Screens Completed (n [%]) 43 [86] Positive Screens (n[%]) 12 [28] Patients Scheduled for Work-up (n [%]) 10 [83] Patients Evaluated (n[%]) 9 [90] Patients Diagnosed with Dementia (n [%]) 3 [25] **Practice F** Patients >75 Years of Age Seen During Intervention Period (n) 138  Screen Forms Initiated (n [%]) 45 [33] Patient Screens Completed (n [%]) 40 [89] Positive Screens (n[%]) 15 [38] Patients Scheduled for Work-up (n [%]) 6 [40] Patients Evaluated (n[%]) 2 [33] Patients Diagnosed with Dementia (n [%]) 0 [0] | I would have to say this: I wonder, really, is diagnosing Alzheimer’s really that big a problem? I mean, how many [patients] do you come up with where you had no idea, you had no suspect [sic] that this person had a problem.... I’ve never seen [medications] be very efﬁcient. (clinician)  ... it would be a lot easier to get enthused about a project that may have more medical relevance. Something that you might really get jazzed about. (clinician)  I thought the study was very useful because it identiﬁed patients that I was not aware of who had problems, whether it was Alzheimer’s or depression or other memory problems. (clinician) | The lack of perceived beneﬁt of diagnosing dementia by some clinicians undoubtedly inﬂuenced their decision whether or not to schedule a memory evaluation. |
| **Boise (1999).** Diagnosing dementia: perspectives of primary care physicians. *Gerontologist*. 1999;39(4):457-464. | Attitudinal barriers included the perception that nothing could be done for patients with dementia, that treatment options were limited or non-existent that neither the patient nor the family would want to have the physician uncover dementia, and that physical problems took precedence over cognitive difficulties.  Time constraints were clearly a significant barrier to the recognition and assessment for dementia, particularly in the urban practices. In rural communities, the primary constraints were the lack of medical diagnostic and community support services.  Constraints in clinical practice, in particular the lack of time, were a serious barrier to recognizing cognitive difficulties in patients. | **Triggers for Awareness of Possible Dementia in Patients Seen by Focus Group Participants (*N* = 18 Groups)** Family brings in patient or raises concern n=16 Problems identified during office visit Memory/thinking problems identified during history taking n=11 Medication problems n=8 Patient symptoms, e.g., weight loss, appearance, difficulty in functioning n=6 Patient self-report n=4 Change in physician results in identification n=2  **Problems identified in health setting outside office visit** Nursing or other staff identify problems n=5 Dementia identified in emergency room, hospital, or nursing home n=4  Missed office visits n=2  **Problem identified in community** Friends, neighbours, or church members contact doctor n=5 Car accidents n=4 Accident or fall at home n=3 Change in patient's living situation, loss of spouse, etc. n=3 Police refer patient n=2 Patient got lost n=2 Workplace referral n=1  **Viewed routine screening for AD**  Either extremely, very or somewhat important Caregivers (97%)  Members of the general public (97%)  Physicians (83%)  Payors (74%) (P≤0.001 for caregivers or general public vs payors or physicians; P≤0.05 for physicians vs payors).  Extremely or very important  Caregivers (84%)  General public (80%)  Physicians (565)  Payors (40%)  Generalists (62%)  Specialists (50%)  (P≤0.05 for the comparison).  **United Kingdom**  Specialists (50%)  Generalists (38%) | I’d say in 90% of the cases it’s the family [that brings the dementia to my attention]. HCP  There's two presentations. The group that comes in worried as heck about their memory, you test them and you can't find any problem. The group that is brought in by relatives, there’s usually a problem. HCP  Sounds like there’s some message coming from somewhere [that doctors should be more] aggressive with early diagnosis...if that's the case that needs to be communicated with some really good reasons. HCP  I don’t want to know, the family doesn't want to know. . . .There [needs to be] something that changes the prognosis HCP  From a screening purpose, to do the test, it takes time. I have 15 minutes. It’s like, why do this Mini-Mental Assessment when the guy is going to score 10. It took me well over 5 minutes to do the test. It took almost half the appointment time to do that. Are you talking about looking for subtle dementia? Everybody over 65 getting a Mini-Mental test? HCP  I’ve walked out of the room lots of times going I think something's going on here but not pushed it, because what am I going to do? What am I going to tell the family? Well, they're functioning okay in the home, I think they've probably got early dementia, but is it going to change anything? No. Can I do anything about it? No. So, why get everybody all excited when we'll just keep a close eye on it. HCP  . . . When we do see people for dementia, it is common that they have ten other medical problems. There's usually something else going on—dementia or memory problems is right at the bottom of the list, in terms of things to address. HCP  I think it depends a lot on if you’re going to approach treatment. You'd put dementia, but you might not put Alzheimer's if you're not going to try any of these things. HCP  I’d probably use dementia until I’m reasonably convinced that it's Alzheimer’s, ruling out other things like depression. HCP  Yes. Probably it’s one of the diagnoses that I have the most trouble discussing with the patients and families. I have no problem about cancer or other fatal diseases but Alzheimer's disease has a huge stigma associated with it. The family doesn't want to hear, the patient doesn't want to hear. The gradual decline of forgetful ness is a much better description to the patient and the family. HCP  I know I’m guilty of it, and suspect the rest of us are too . . . which is that, with someone who’s been your patient for a while and, you haven’t done a MiniMental State Exam on them. It's very easy to talk to people with dementia and say, Hi, how ya doin? Great. Feeling good today? Yeah. How's that blood pressure medicine working? Good. You taking it the right way? Yeah, just the way you told me, doc. Then, you give them a Mini-Mental State [Examination], and you go Oh my goodness. That could be very easy to miss. HCP  My role as a physician is trying to help the family deal with [the dementia]. The patient, other than maybe adjusting, a psychotropic to try to control their outburst, things like that, is actually fairly simple. It's dealing with the social problems. HCP  Since we don't have any really good medications, you say, Is this a particular kind of dementia, multi-infarct versus Alzheimer's? I don't do a whole lot past the metabolic [lab tests]. I haven't thus far. . . . I'm not convinced we can do a whole lot for those people and you have to question: Is going past that worth our time and effort? | Until there are effective treatments, there is little reason to assess patients for cognitive problems |
| **Bond (2010).** Screening for cognitive impairment, Alzheimer's disease and other dementias: opinions of European caregivers, payors, physicians and the general public. *J Nutr Health Aging.* 2010;14(7):558-562. | The most frequently cited reasons given by physicians who did not favour routine screening at age 65 were screening inaccuracy, costs and lack of treatment. Most cited by the general public for not routinely screening were screening inaccuracy, costs and time. For caregivers, they were screening inaccuracy, nothing you can do if you know and monetary cost.  This paper argued that the barrier of no effective treatment often cited may be debated, since treatments to reduce the decline in cognition, and the associated loss of function, could have benefits at the earliest evidence of dementia  No generally acceptable test exists for use in population screening although there are a number of recommendations for use in primary care case finding  A larger proportion of caregivers and members of the general public viewed routine screening for AD as extremely or very important | **Respondents, % Reason for not screening**  **Physicians**  I. Screenings for AD are not accurate 44 II. It will cost too much money 33 III. There is no treatment for it 24 IV. It takes too much time 19 V. There is nothing you can do if you know 12 VI. It would be depressing to know 12 VII. It is not necessary to detect early 11 VIII. Other reason 18 **Caregivers**  I. Screenings for AD are not accurate 31  II. It will cost too much money 28  III. There is no treatment for it 17 IV. It takes too much time 10  V. There is nothing you can do if you know 31 VI. It would be depressing to know 10  VII. It is not necessary to detect early 10  VIII. Other reason 17 **General public** I. Screenings for AD are not accurate 29 II. It will cost too much money 19 III. There is no treatment for it 11 IV. It takes too much time 16 V. There is nothing you can do if you know 9 VI. It would be depressing to know 13 VII. It is not necessary to detect early 8 VIII. Other reason 25 **Should everyone should be routinely screened for AD at age 65**  Physicians (42%)  Payors (44%)  General public (81%)  Caregivers (80%)  (P<0.001 for caregivers or general public vs physicians or payors). | General public may have a more positive opinion of routine screening for AD than those physicians or payors who responded.  The most frequently cited reason given by those who did not favour routine screening at age 65 was screening inaccuracy. P≤0.001). In the United Kingdom more specialists than generalists (50% vs 38%) agreed screening tests are not accurate. | The most frequently cited reason for physicians from the United Kingdom (44%), was screening inaccuracy. In the United Kingdom were there more specialists (34%) than generalists (22%) who favoured routine screening.  Relevant physician action occurred in only 17% of screen-positive patients |
| **Borson (2007).** Implementing routine cognitive screening of older adults in primary care: process and impact on physician behavior. *J Gen Intern Med.* 2007;22(6):811-817. | Acceptability of screening for both staff and patients was high, based on very low patient refusal rates and few complaints by medical assistants however the departure of the staff champion, whose role was to identify patients eligible for screening and to oversee the process, compromised the completion of the intervention.  MAs and physicians made few complaints about the screening intervention, and their informally reported perception of workflow was not significantly affected by the use of the short screen adopted for this study and the related charting processes. | **Screened positive**  Patients (n=748), 524 (70%) were screened and 18% screened positive. Less than 1% of eligible patients refused and 4% were not screened for reasons specified by MAs (refusal, acute medical illness, non-English speaking, blindness, deafness). 26% of eligible patients were not screened with no reason reported **Dementia diagnoses**  Intervention  Geriatricians increased 3.9% (39 to 53 cases)  Non-geriatricians increased 1.5% (17 to 23)  Controls increased 0.5% (30 to 35)  **Dementia specialty referrals**  Geriatricians increased 0.3% (3 to 4)  Non-geriatricians increased 1.5% (3 to 10)  Controls increased 0.1% (5 to 6)  Significant increase by non-geriatricians (F=7.1, P= 0.008, df=1, 360), control clinics (F=10.8, P=0.001, df=1, 1,553).  **Dementia medication prescriptions**  Geriatricians did not change (0%, 18)  Non-geriatricians increased 1.2% (6 to 11) Controls increased 0.35% (18 to 22) **Proportion of patients with diagnosed dementia** Increased in both control and intervention clinics and for both geriatricians and non-geriatricians (F= 14.52, P=0.000, df=1, 360, for geriatricians’ patients; F=6.08, P=0.014, df=1, 414 for intervention clinic non-geriatricians’ patients; F=6.03, P=0.014, df=1, 1,139 for control clinics).  Patients with positive screens were significantly more likely than all other groups to receive a new dementia diagnosis (P<0.01) or specialty referral (P<0.01). | 26% of patients eligible for screening were not screened, with no specific reason recorded by the responsible MAs or the supervising RN. There could be many reasons for this, including aspects of clinic dynamics, individual MA factors, and patient factors. The data required to address all of the possible reasons why screening did not occur for 26% of eligible patients are relevant but unfortunately were not collected as part of this study.  It appears that patients have concerns about dementia screening such as losing their health insurance coverage, their driver privileges, and/or their employment positions.  Relative to controls and negative screens, positive screens were associated with higher rates of dementia-related physician actions.  The magnitude of physician responses to screening as an isolated practice intervention, while statistically significant, was small  Primary care physicians acted mainly on positive screens when cognitive impairment was severe and only when patients were 75 years old or older. | Relevant physician action occurred in only 17% of screen-positive patients.  Patient’s refusal to proceed with a diagnostic evaluation following a positive screening test. Nearly half of the patients who screened positive for cognitive impairment refused a diagnostic evaluation. One reason for these refusals may be the patient’s perception of the harm of dementia screening  Patients with positive screens were significantly more likely than all other groups to receive a new dementia diagnosis (P<0.01) or specialty referral (P<0.01). |
| **Boustani (2011).**  Caregiver and noncaregiver attitudes toward dementia screening. *J Am Geriatr Soc.* 2011;59(4):681-686 | A complex interaction of perceived harms and beneﬁts that made it difﬁcult to determine whether the beneﬁts of dementia screening outweighed the potential harms | **Acceptance score for screening**  Caregiver vs noncaregivers (53.9 vs 60.6; P=.02).  **Top ﬁve barriers**  Perceived suffering and perceived effect on independence; 75% to 86% felt they would suffer emotionally, 75% to 78% were concerned they would lose their driving license and other privileges, 43% to 64% felt they would be depressed, 45% to 59% were concerned they would suffer ﬁnancially, and 37% to 51% felt they would be anxious. **Independence score**  Significantly lower for caregivers (47.6 vs 54.0; P5.004)  **Suffering score** Caregivers had a significantly higher mean (61.6 vs 55.9; P5.02)  **Beneﬁts of the dementia screening**  Caregivers significantly higher mean score (72.8 vs 69.0; P5.03). | Caregivers and non-caregivers generally disagreed that the perceived stigma would be a consequence of dementia with caregivers scoring lower. | A complex interaction of perceived harms and beneﬁts that made it difﬁcult to determine whether the beneﬁts of dementia screening outweighed the potential harms |
| **Boustani (2008).**  Measuring primary care patients' attitudes about dementia screening. *Int J Geriatr Psychiatry.* 2008;23(8):812-820. | Major barriers to cognitive screening were lack of time, risk of offending patients, and possible negative consequences of follow-up. Clock drawing was perceived as an acceptable method of screening, if it were proven effective. | The screen plot suggested the existence of one or two factors. Clinical judgment suggested that the two-factor solution was sensible. Found that the acceptance of screening on two dimensions, accepting the screening to know the risk and accepting the actual testing for AD screening. | Having more time for future planning for an individual’s health, ﬁnancial, and housing needs and more time for an overall family discussion and planning. Both of these perceived beneﬁts and harms are inﬂuencing the patient’s decision of accepting dementia screening. This decision in the current data is perceived favourably for the general screening method. | Physicians are receptive to using the clock drawing test, and, because it is not time-consuming, are less likely to consider lack of time a barrier to testing. The clock test might help bridge the gap between perceived need for screening and actual screening. |
| **Boustani (2003).** Acceptance of dementia screening in continuous care retirement communities: a mailed survey. *Intl J Geriatr Psychiatry*. 2003;18(9):780-786. | 49% of participants would agree to routine screening for memory problems.   Older adults had concerns about routine dementia screening by cognitive questionnaire.   Study did not explore the reasons that residents were unwilling to be screened regularly for dementia | **(N %) [willing to be screened for memory problems] p-Value** Female (215) [46.7] 0.363; Male (103) [53.0] Married (182) [45.6] 0.195;  Not married (136) [52.9] Living alone (170) [52.9] 0.108;  Not living alone (148) [43.9] 0–1 Comorbidities (118) [39.0] 0.008;  2+ Comorbidities (200) [54.5] Hearing or vision problem (126) [56.4] 0.028;  No hearing or vision problem (192) [43.8] Less than 3 medications (68) [30.9] 0.001;  3+ medications (250) [53.6] Use device or system to remember meds (159) [60.4] <0.001;  Does not use device or system to take meds (159) [37.1] Any memory problem (24) [58.3] 0.328;  No memory problem (294) [48.0] Problem with meds (25) [52.0] 0.734;  No problem with meds (993) [48.5] **Associations between dementia screening acceptance and selected variables, adjusted for covariates OR (95% CI) p-Value** Female 0.53 (0.31, 0.93) 0.028; Married 0.65 (0.33, 1.30) 0.228 Living alone 0.90 (0.44, 1.81) 0.756  Hearing or vision problem 1.19 (0.70, 2.02) 0.512 2 or more comorbidities 1.26 (0.71, 2.26) 0.431 Any memory problem 1.34 (0.55, 3.26) 0.522 Problem with meds 0.58 (0.24, 1.41) 0.233 Any depressive symptoms 1.17 (0.58, 2.36) 0.672 No device for meds,<3 meds 1.00; Use device for meds,<3 meds 10.45 (2.74, 39.88) 0.001; No device for meds, 3 or more meds 2.99 (1.30, 6.89) 0.010; Use device for meds, 3 or more meds 5.98 (2.54, 14.09) <0.001 | Approximately half of the residents in this afﬂuent residential community setting were not willing to be screened routinely for memory problems.  Those using assistive devices or a  system to help them take their medications were more willing to accept dementia screening than those who did not use assistance.   Individual acceptance of dementia screening could be a reﬂection of their attitude toward screening for asymptomatic medical conditions in general, not speciﬁcally dementia; healthy individuals may not seek medical assessment for any asymptomatic conditions. | Almost half of this largely white, afﬂuent sample said they would not want to be screened for memory impairment on a regular basis. Their willingness to be screened for dementia was strongly associated with their acceptance of depression screening and being male; high refusal rate indicates that dementia screening may be associated with perceived harms.  Types of cognitive difﬁculties were also associated with more willingness to be screened for dementia. However specific types of cognitive difﬁculties were not described by study author.  Possible discrimination due to insurers and employers gaining access to screening results. |
| **Brodaty (1994).**  General practice and dementia. A national survey of Australian GPs. *Med J Aust.* 1994;160(1):10-14. | Evidence suggests that general practitioner rely on a passive approach (39%) to screening for dementia. | 20% GPs regarded *providing a patient with a diagnosis of dementia as usually more harmful than helpful*  39.2% reported conducting *regular screening for cognitive deficits in their elderly patients* 33% indicated that they would *wait and observe for signs of deterioration* 22% indicated they would *refer immediately for specialist assessment* 10% of GPs *never or rarely* referred patients to Geriatric or Aged Care Assessment Teams  11.1% of GPs never or rarely referred patients to Aged Care Community Services  5.9% there is *no advantage in early diagnosis* | It is often difficult to diagnose without family input especially in the early stages; often patients when attending the surgery appear to be relatively normal and the GP may be unaware of other patients who are not attending  Because the absence of any treatment the exact point of time of diagnosis is not important  Poor prognosis...sense of uselessness | Respondents supported five structural modifications:  - a protocol for assessment and investigation (92.6%)  - brief screening instrument for early detection (89.9%)  - community based geriatric nurse (86.4%)  - central register of residential facilities (82.9%)  - Medicare team for consultations (76.6%)  The implementation of any screening programme would involve training GPs, particularly in early diagnosis. Merely offering educational programs to all GPs is unlikely to address this successfully as many do not regard dementia as a high priority and others would find it difficult to attend sessions. |
| **Bush (1997).** Screening for cognitive impairment in the elderly. *Can Fam Physician.* 1997;43:1763-1768. | 287 (82.2%) of the sample believed in a need to screen (95% CI, 78.2% to 86.2%) however only 24% HCPs asked routinely screened patients   Three hundred forty-seven (94.8%) respondents believed that cognitive impairment affects medical management (95% CI, 92.7% to 97.3%).   260 (74.3%) reported no routine cognitive screening of their elderly patients (95% CI, 69.7% to 78.9%);  On average, younger physicians and those in a group practice indicated an ideal time that was 3 min shorter than older physicians and those in solo practice.   Validity/accuracy of screening tool was deemed the top attribute for primary care physicians, followed closely by ease of administration and time required. | **Reasons for not screening elderly patients for cognitive impairment: N. Respondents [reason (%)] CI intervals (95%)** Should assess cognition only if suspicions aroused by other evidence 32 (51) 33-68 No proven benefit 12 (19) 0-41 Usually obvious without screening 8 (13) 0-36 Inefficient or not cost-effective 6 (9) 0-32 Other reasons 18 (29) 8-50 Reason unclear 10 (16) 0-39  **Perceived barriers to screening for cognitive impairment: N. Respondents [barrier (%)] CI intervals (95%)** Lack of time 288 (85) 81-89 Patients offended or resistant 197 (58) 51-65 Negative consequences of follow up 82 (24) 15-33 Lack of proven benefit 76 (22) 13-31 Available tests inadequate 74 (22) 13-31 Other 31(9) 0-19 | Groups agreed on the benefits and risks of early diagnosis of dementia; disagreed about screening for dementia, and about professional resistance to making the diagnosis; constructed comprehensive guidelines on diagnosis, but without much reference to resource implications; yet described actual local resource limitations in detail; and avoided dilemmas about dementia care by framing it as a specialist activity. | This result reﬂects the complexity of screening in situations where the effectiveness of medical treatment for conditions such as dementia is modest at best. |
| **Cahill (2008).** The attitudes and practices of general practitioners regarding dementia diagnosis in Ireland. . *Int J Geriatr Psychiatry.* 2008;23(7):663-669. | GPs were more likely to blame themselves than the health care system, their patients or family members for the late presentation of dementia in primary care. Stigma was a major obstacle preventing GPs from being more proactive in this area. Rural GPs felt geographically disadvantaged accessing diagnostic services and both rural and urban GPs experienced considerable time delays accessing specialist diagnostic services. | **Time delays in accessing specialist geriatric and psycho-geriatric services for GPs** Psychiatry of Old Age (OAP) (n=293) Immediate Access 43%  Wait times>3 months but<1 year 37%  Wait time>1 year 4%  No Access Publicly 15%  Don’t Know 1%  Geriatric Medicine (GM) (n=292) Immediate Access 41% Wait times>3 months but <1 year 55%  Wait time>1 year 3%  No Access Publicly 1%  Don’t Know 0   Neuro-Psychologist (NP) (n=286) Immediate Access 1% Wait times>3 months but<1 year 11% Wait time>1 year 9% No Access Publicly 76% Don’t Know 3% | ‘Very often you know these patients very, very well and have seen them over many years ... and maybe you don’t notice, because of your lack of memory ... their lack of memory and then it’s really only when a crisis occurs, you know, something happens that sort of makes everybody stand back and say ‘Oh my God, it’s really obvious’ and made it difﬁcult for most to administer the MMSE’   ‘The MMSE is quite distressing...to do with a patient you know, I think it’s quite an invasive test...I think part of the problem is that the minute you start doing it, it’s ...very direct ...’  ‘I think that the label is the problem. I think all of us (GPs) are reluctant to label somebody because that immediately puts them in a category of high dependency...so I think all of us...you know, we’ll muddle along in a grey area until...it’s clear a patient can’t manage on their own ...’  ‘I think it wouldn’t be so much getting paid for doing the tests, ... it may be much more successful to have some sort of a scheme whereby we were taught to really look after them [patients] and are paid to really look after them .. .’  ‘There is an absence of worthwhile treatment’  ‘Lack of urgency as early diagnosis has little effect on the progress’.   ‘Lack of beneﬁt in knowing earlier’.   ‘There is no proven advantage to early diagnosis'.  ‘Screening symptoms are missed during short consultation’;  ‘There is a lack of training in dementia diagnosis’  ‘Patients are embarrassed to admit to memory problems’.   ‘Patients also present late because they feel there is no treatment’. | Half (55%) the GPs waited a minimum of 3 months for GM consultation, more than a third for OAP services and for both services, immediate access was uncommon. Access to NP services was even rarer. Such time delays may further disadvantage patients and their families, since clinical treatments and access to some community services often require more specialist diagnostic input.  A small minority (n=17) saw no value whatsoever in early diagnosis: ‘lack of urgency as early diagnosis has little effect on the progress’, ‘lack of beneﬁt in knowing earlier’, ‘there is no proven advantage to early diagnosis’ |
| **Carpenter (2011).** Physician and nurse acceptance of technicians to screen for geriatric syndromes in the emergency department. *West J Emerg Med.* 2011;12(4):489-495. | Most nurses and physicians identified geriatric technicians as beneficial to patients without impeding ED throughput. Fewer than 25% of physicians routinely screen for any geriatric syndromes. Nurses evaluated for fall risk significantly more often than physicians, but no other significant differences were noted in ongoing screening efforts.  Few physicians or nurses identiﬁed barriers to geriatric  technician screening for geriatric syndromes in the ED.  Most respondents believed that geriatric technician screening would have a positive effect on patient care and patient safety, but one third were neutral regarding the level of difﬁculty in implementing this clinical strategy. | **Physician and nurse assessment of geriatric technician screening upon patient care** Very positive n=25 45% Somewhat positive n=16 29% Neutral n=12 22% Somewhat negative n=1 2% Very negative n=1 2% **Physician and nurse assessment of geriatric technician screening upon patient safety** Very positive n=27 49% Somewhat positive n=19 34% Neutral n=8 15% Somewhat negative n=0 0% Very negative n=1 2% **Physician and nurse assessment of difficulty in implementing a geriatric technician screening program** Not difficult n=7 13% Slightly difficult n=14 26% Neutral n=19 34% Somewhat difficult n=5 9% Extremely difficult n=10 18% **Perspectives on geriatric syndrome screening in emergency department n(%)** Physicians (n=21)  Outside the realm of emergency medicine 1 (5)  Screening already performed 0 (0)  Screening will delay ED throughout 1 (5)  Screening will not improve outcomes 1 (5)  Cost prohibitive 0 (0) Screening too invasive for patients 0 (0) Nurses (n=34)  Outside the realm of emergency medicine 1 (3)  Screening already performed 1 (3)  Screening will delay ED throughput 2 (6)  Screening will not improve outcomes 0 (0)  Cost prohibitive 1 (3)  Screening too invasive for patients 0 (0) **Current geriatric screening in emergency department without geriatric technician, using validated tools** Physician (n=21)  Dementia, No. (%; 95% CI) 5 (24; 6–42)  Functional status, No. (%; 95% CI) 5 (24; 6–42) Nurses (n=34)  Dementia, No. (%; 95% CI) 7 (21; 7–34)  Functional status, No. (%; 95% CI) 10 (29; 14–45) | ‘Screening symptoms are missed during short consultation’  ‘There is an absence of worthwhile treatment’ | While most physicians and nurses believed that a geriatric technician would enhance patient safety and improve patient care, 35% of nurses and 19% of physicians believe that such a role would be difﬁcult to implement in most EDs.  Fewer than 25% of physicians routinely screen for any geriatric syndromes.   Most nurses and physicians do not believe that screening older adults for prevalent geriatric syndromes is outside the realm of EM. |
| **Dale (2006).**  High interest in screening and treatment for mild cognitive impairment in older adults: A pilot study. *J Am Geriatr Soc.* 2006;54(9):1388-1394. | Ninety-eight percent of those healthy adults who responded would be willing to be tested for MCI if a family member suggested they had memory problems | **Attitudes Toward Alzheimer’s Disease** Do you worry about developing Alzheimer’s disease?  Definitely Yes n=15  Probably Yes n=26  Definitely No n=17  Probably No n=41  Do you feel you are more likely to develop Alzheimer’s disease over your lifetime than most? Definitely Yes n=6  Probably Yes n=13  Definitely No n=39  Probably No n=41  Would you be surprised if you developed Alzheimer’s disease in your lifetime? Definitely Yes n=35  Probably Yes n=21  Definitely No n=27  Probably No n=16  Would you want to know as early as possible that you had Alzheimer’s disease? Definitely Yes n=82  Probably Yes n=10  Definitely No n=5  Probably No n=4 | Those saying that they would want to know early if they had AD were significantly more willing to be tested and treated for MCI across the board.  Those who had previously heard of MCI were much less willing to be tested (38% vs 63%; P5.05).  Those who believed that they were more likely than average to develop AD and those who had relatives with AD were much more willing to be tested if a family member suggested testing. | Although the evidence supporting the combined use of cholinesterase inhibitors and multicomponent interventions to treat early AD to delay nursing home placement may justify identiﬁcation of those with early AD, it does not currently justify population screening for early AD.  One attitude made people more desirous of screening and treatment for MCI: wanting to know early if one had AD.  A conclusion of this paper was that enthusiasm for screening is unwarranted given the paucity of evidence. |
| **Dale (2008).**  What correlates with the intention to be tested for mild cognitive impairment (MCI) in healthy older adults? *Alzheimer Dis Assoc Disord.* 2008;22(2):144-152.. | Over 60% of the participants do not worry about AD and over 80% do not believe they are more likely to get it than others, over 80% said they would deﬁnitely want to know early if they have AD.  Over 80% knew someone with AD, with nearly half having a relative with AD, and 40% having cared for someone with AD. Although 40% of the participants believe they currently have memory problems, 15% believe they always have had such problems. Only about 15% have had previous memory testing. | Those who were most likely to say they ‘‘want to know as early as possible if they have AD’’ were more interested in receiving this type of screening for MCI, and this was the only predictor signiﬁcant at the P<0.01 level.  Four additional predictors were signiﬁcant at the P<0.05 level; younger age, thinking one currently has a memory problem, having a chance-oriented LOC and higher depressive symptoms. | NA | Apprehension over a possible AD diagnosis potentially resulting from further testing contributes to the lack of follow-up.  Younger age, thinking one currently has a memory problem, having a chance-oriented LOC and higher depressive symptoms all impact on wanting an assessment  The strongest predictor is a desire to know as early as possible if one has AD. |
| **Downs (2000).**  Caring for patients with dementia: The GP perspective. Aging*Ment Health.* 2000;4(4):301-304. | GPs were unlikely to report suspecting dementia following regular screening for cognitive impairment.  Nearly all of the GPs in our study stated that they rarely consider the use of dementia speciﬁc medication as a sufﬁcient reason to actively screen for dementia among their older patients. Several of the GPs linked this reluctance to their concerns about treating what they viewed as a ‘normal’ problem related to the ageing process.  Few (15%) reported that they were led to suspect dementia following regular screening for memory loss in their elderly patients. Neither age, gender nor postgraduate training distinguished those who reported conducting regular screening from those who did not. Eighty per cent of GPs reported that they conducted medical tests to exclude an underlying cause. | **Percentage of GPs reporting difficulty with various aspects of dementia care** Diagnosis issues Establishing a definitive diagnosis 48% Disclosing diagnosis to patient 41% Disclosing diagnosis to family member 21%  Treatment Responding to behaviour problems 74% Responding to patient’s social problems 58%  Administering medications 48% Responding to families’ concerns 44% Responding to patient’s psychiatric problems 37%  Support services Co-ordinating support services 59% Getting information about support services 55%  **Percentage of GPs reporting what may help them better respond to people with dementia and their families** Assessment protocol 84% Screening instrument 78% Information about available support services 76% Contact details about support services 72% Geriatric nurse attached to practice 71% Information about old age psychiatry services 54% Register of residential facilities 49% Information about housing 45% | The drugs that we’ve been promised might be useful are not that brilliant, so you do wonder why you bother at times trying to pick it early (GP 10).  I’m interested in trying to let people live a normal life and not become a patient. The problem is that we are medicalising the ageing process, and some of my specialist colleagues want to put people on too many medications (GP 13). | Few GPs reported that they referred people with dementia and their families to Alzheimer’s Scotland- Action on Dementia.  A substantial majority responded that an assessment protocol (84%) and a screening instrument (78%) would be of help. Almost three-quarters (71%) agreed that having a geriatric nurse attached to the practice would help. |
| **Fowler (2012).**  Effect of patient perceptions on dementia screening in primary care. *J Am Geriatr Soc.* 2012;60(6):1037-1044. | After adjusting for age, perception of depression screening, perception of colon cancer screening, and belief that no treatment is currently available for Alzheimer’s disease, the odds of refusing screening were signiﬁcantly lower in participants who had higher PRISM-PC domain scores for beneﬁts of dementia screening (odds ratio (OR) = 0.85, 95% conﬁdence interval (CI) = 0.75–0.97; P = .02). In the same regression model, the odds of refusing screening were signiﬁcantly higher in participants aged 70 to 74 (OR = 5.65, 95% CI = 2.27–14.09; P < .001) and those aged 75 to 79 (OR = 3.63, 95% CI = 1.32–9.99; P = .01) than in the reference group of patients aged 65 to 69. | **Logistic Regression Analysis of the Odds of Refusing to Undergo Screening for Dementia** *Odds Ratio (95% CI) PValue* High domain score: perception that dementia screening is beneficial 0.85 (0.75–0.97) .02 Belief that no treatment is currently available for Alzheimer’s disease 1.29 (0.93–1.78) .13  **Bivariate Comparison of Mean PRISM-PC Scores of Study Participants Who Accepted and Refused Screening for Dementia** **Mean Score ± Standard Deviation** Accepted Screening, n = 497 Benefits of dementia screening 73.0 ± 10.5  Stigma of dementia screening 33.1 ± 12.2  Negative impact of dementia screening on independence 50.3 ± 13.9  Suffering related to dementia screening 52.9 ± 15.2  Refused Screening, n = 57 Benefits of dementia screening 67.3 ± 12.4  Stigma of dementia screening 33.0 ± 11.5  Negative impact of dementia screening on independence 48.4 ± 13.8  Suffering related to dementia screening 52.7 ± 14.3  P Value Benefits of dementia screening .001 Stigma of dementia screening .95 Negative impact of dementia screening on independence .33 Suffering related to dementia screening .88 Accepted Screening Belief that a treatment for Alzheimer’s disease is not currently available 2.7 ± 0.9  Refused Screening Belief that a treatment for Alzheimer’s disease is not currently available 3.0 ± 0.8 P Value Belief that a treatment for Alzheimer’s disease is not currently available.02  **Bivariate Comparison of Mean PRISM-PC Scores of Study Participants Who Screened Positive and Screened Negative for Dementia** **Mean Score ± Standard Deviation** Screened Positive, n = 63 Benefits of dementia screening 71.7 (10.7)  Stigma of dementia screening 39.8 (12.7)  Negative effect of dementia screening on independence 50.4 (15.6)  Suffering related to dementia screening 51.2 (16.6)  Screened Negative, n = 434 Benefits of dementia screening 73.1 (10.4)  Stigma of dementia screening 32.1 (11.8)  Negative effect of dementia screening on independence 50.3 (13.7)  Suffering related to dementia screening 53.1 (15.0)  PValue Benefits of dementia screening .30 Stigma of dementia screening <.001 Negative effect of dementia screening on independence .96 Suffering related to dementia screening .35 Belief that a treatment for Alzheimer’s disease is not currently available Screened Positive 2.8 (0.9) Screened Negative 2.7 (0.9) PValue .43 | Participants in the middle age range of the sample (70–79) were less likely to accept screening for dementia than younger (65–69) and older participants (<80). | Only age was highly predictive of acceptance |
| **Galvin (2012).**  Improving physician awareness of Alzheimer’s disease and enhancing recruitment: The clinician partners program. *Alzheimer Dis Assoc Disord.* 2012;26(1):61-67. | Post-tests showed improvements in knowledge and confidence to diagnose and treat and increased use of dementia screening tools.  Able to change practice habits as evidenced by the incorporation of dementia screening tools such as the Short Blessed Test22 and the AD814 into the CPP graduates’ evaluations of older adults. | Notably, 98% of attendees agreed that they had learned new tools from the training that would enhance their clinical practice. CPP graduates reported increased use of dementia screening tools in their practice at the 3-month and 12-month post-test  At the time of the pre-test, 13% of clinicians were not using any dementia screening tools in their practice, however by the 12month post-test, all CPP graduates who completed the post-test were using at least 1 dementia screening tool and 66% were using a combination of 2 or more instruments. | CPP graduates were also asked to describe changes in their practice at the post-test periods in open-ended responses. Comments ranged from better recognition of dementia symptoms to more comfort with treatment modalities and increased awareness of community resources. | Identified the combination of a performance test such as the Short Blessed Test and an informant questionnaire such as the AD814 useful.  many physicians remain unaware of useful services in the community |
| **Galvin (2008).**  Psychosocial determinants of intention to screen for Alzheimer's disease. *Alzheimers Dement.* 2008;4(5):353-360. | Predictors of intention to screen included perceived beneﬁts (knowledge of dementia, self-efﬁcacy, preventive health behaviours, and perceived susceptibility. | **Predictor of intention**  Perceived beneﬁts (ɣ.35)  knowledge of dementia (ɣ.26)  self-efﬁcacy (ɣ .23)  health behaviours (ɣ.17)  perceived susceptibility (ɣ.14)  Knowledge and consequences of dementia positively correlated with perceived beneﬁts (φ .20). Self-efﬁcacy positively correlated with preventive behaviours (φ.37). Preventive health behaviour correlated with perceived beneﬁts (φ= -.11) and negatively correlated with self-efﬁcacy. φ .29) and perceived susceptibility ( φ .20) and perceived beneﬁts (φ - .20); perceived susceptibility φ .26), self-efﬁcacy (ɣ.23)  From 2006 paper: Dementia knowledge  Satorra-Bentler Scaled X2 (P Value) -  RMSEA - α 0.26  Screening knowledge  Satorra-Bentler Scaled X2 (P Value) 80.38 (<0.01) RMSEA 0.04 α 0.71  Perceived barriers (to screening)  Satorra-Bentler Scaled X2 (P Value) 24.98 (0.25) RMSEA 0.02 α 0.70  Perceived benefits (of screening)  Satorra-Bentler Scaled X2 (P Value) 7.99 (0.02) RMSEA 0.06 α 0.75 | NA | Predisposing factors (particularly knowledge and perceived beneﬁts) might be more important than enabling and need factors in promoting the screening for dementia among older adults. |
| **Hansen (2008).**  General practitioners’ experiences and understandings of diagnosing dementia: factors impacting on early diagnosis. *Soc Sci Med.* 2008;67(11):1776-1783.  . | Key ﬁndings are that dementia is a complex condition that takes time to diagnose. Diagnosis may involve conﬂict between GPs, family members/carers and the person with dementia (PWD). GPs did not consider that diagnosing dementia early was particularly important and may in fact be harmful to some patients. They are sceptical about the advantages of dementia medications. GPs assess the need for a formal diagnosis of dementia within the broader context of their older patients’ lives. They are more likely to pursue a formal diagnosis in situations where they see it leading to beneﬁts for their patient such as accessing dementia speciﬁc services. | NA | Very difﬁcult to pick in an average general practice. You tend to see people for 15–20 minutes and you are usually dealing with physical problems and not the cognitive or ﬁner points of people’s behaviour (GP 5)  I ﬁnd that if I see a patient in my rooms it [dementia] may not be that obvious, they present OK..but if you talk to their sister or their daughter you get a different story, so I ﬁnd that’s a much better way to pick up problems (GP 10)  You are not always dealing with just dementia, you are often dealing with elderly frail people so I don’t suppose the dementia diagnosis as such would make much difference . I would usually see dementia in a spectrum of conditions that an elderly person might have (GP 13)  I think most of us GPs wait until something happens before you do anything. .I’ve had several people who are severely demented that I didn’t know were demented until their elderly husband or wife died (GP 13)  There’s so little that one can do that realistically makes a difference in terms of outcomes. I’m sure that if you can utilize them [support services] to the maximum there’s a hell of a lot that can be done. But as far as altering the course of the disease, no (GP 12)  I can’t go and steamroll your mother into taking a test that she will have enough insight to realize is a direct attack on her ability to keep coping (GP 14).  I guess ageing people never really complain of dementing processes because dementia is almost euphoric, you tend to live with it rather than suffer from it. Whereas ‘Oh gosh is my Mum dementing?’ that comes from a child (GP 1).  The drugs that we’ve been promised might be useful are not that brilliant, so you do wonder why you bother at times trying to pick it early (GP 10).  If they’re suitable for medication then it hopefully will prevent it [dementia] progressing or allow it to progress more slowly, and if they have enough insight into what’s going on then they make plans for the future and talk about it (GP 7).  On the opposite side, it [early diagnosis] can be detrimental to their general wellbeing because most of them don’t want a diagnosis of dementia. And if they aware enough to say ‘I don’t want that’, then they could end up with depression (GP 7). | The reluctance of rural GPs to make use of psycho-geriatricians reflects a more autonomous approach to their practice and the lack of access to such services in rural areas.  a dearth of available places in residential aged care and GP perception of a lack of time to deal with complicated problems in their patients due to doctor shortages |
| **Holsinger (2011).** Acceptability of dementia screening in primary care patients. International *Int J Geriatr Psychiatry.* 2011;26(4):373-379. | Overall, 81% of primary care patients indicated that they would want to be screened to determine if they are developing dementia. After exposure to possible risks and beneﬁts of screening, 86% of patients indicated they would like to be screened. | **Factor analysis summary** Cronbach’s α Prior experience with dementia 0.51 Acceptance of screening for dementia 0.92 Current treatment for dementia 0.77  **Unadjusted odds ratios of screening acceptance (95% CI)** Other screening acceptance 4.3 (2.7 6.7) Gender (males more accepting of screening) 3.3 (1.9 5.9) VA patient (versus Duke patient) 3.1 (1.8 5.6) Belief that treatment exists 2.6 (1.6 4.2) Desire to use time for planning 2.1 (1.3 3.7) Younger age (OR per decade) 1.6 (1.3 2.2) Subjective memory complaints 3.6 (1.3 7.7)  74% would ﬁnd a questionnaire acceptable, 68% would agree to a blood test, and 51% would agree to neuroimaging.  Three explanatory variables associated with dementia screening acceptance: acceptance of other screens (OR: 3.7, CI: 1.9, 7.1), male gender (OR: 3.16, CI: 1.2, 7.8), and a belief that treatment for dementia exists (OR: 2.24, CI: 1.09, 4.59). The remaining variables with signiﬁcant unadjusted odds ratios dropped below the level of signiﬁcant association in the adjusted analysis. | NA | Subjects may have had unrealistically optimistic beliefs about the beneﬁts of current treatments, but they did demonstrate concern about possible adverse effects of a dementia diagnosis.   Screening tests for dementias do not provide a diagnosis, and the necessary components of an evaluation must be available for those who screen positive  Several hurdles remain: uncertainty regarding future healthcare coverage with a new dementia diagnosis, and clinician perception. |
| **Iliffe (1994).** Evaluation of the use of brief screening instruments for dementia, depression and problem drinking among elderly people in general practice. *Br J Gen Pract.* 1994;44(388):503-507 | Although screening instruments were not completed in 14% of the random sample, many of the individuals for whom screening results were not obtained were seriously ill, and their general practitioners felt administration of the screening instruments would have been detrimental to patient care. | When the general practitioners were using clinical judgement alone practice A were significantly more likely to diagnose dementia (Fisher exact test, P<0.05) or depression (X^2^ test, P<0.001) than doctors in practice B. | In most cases the doctors felt that the individual was too ill to proceed with a full assessment or to use screening instruments, although lack of time was also given as a reason. | The observed prevalence of possible dementia was higher than the expected prevalence of 6-12% found in community studies of similar populations in north London, but this is probably because the instruments were used opportunistically in a population consulting its doctors.  Assessment of the health of elderly patients can use brief screening instruments for dementia and depression as the first step in case finding for these common conditions, but if the general practitioners in this study are typical of the profession, the case for doing so remains unproven.  The promotion of psychiatric screening instruments in assessment packages like that published by the Royal College of General Practitioners may be premature, and larger scale studies of the outcome of using such instruments are needed to establish their true value. Trials of different approaches to treating depression, and of different ways of caring for people with dementia in the community, would be especially useful. |
| **Iliffe (2003).**  Sooner or later? Issues in the early diagnosis of dementia in general practice: a qualitative study. *Fam Pract.* 2003;20(4):376-381. | Two workshops made explicit comments about the ethics of screening, the problems of obtaining informed consent, the threat of screening to the psychological stability of patients and the problem of overcoming taboos about dementia to allow screening to be accepted.  There was no agreement between workshops about whether the new anti-dementia medication meant that early treatment had become more effective than late treatment, nor was there agreement on a suitable test. | NA | “The advantages of promoting early recognition of dementia in the community including shifting budgets—we will need more physiotherapists, district nurses, community psychiatric nurses, occupational therapists and nursing home places to respond to an ageing population.”  “Would not tell anyone they are unsuitable for treatment, but would refer them to social services and the Alzheimer’s Society.”  “The majority (of professionals) do not and have no reason to resist”.   “Insight into the diagnosis is almost a relief but until recently nothing could be done. Now (dementia) is worthwhile to diagnose”   “Relatives feel that there may be a treatment and a cure in the future”.  No workshop used the terms ‘case finding’ or ‘assessment’ as alternatives to ‘screening’, when discussing individual rather than population perspectives. | Term diagnosis could usefully be replaced by recognition to aid a shift in model  Promoting early recognition of dementia in the community including shifting budgets, more physiotherapists, district nurses, community psychiatric nurses, occupational therapists and nursing home places to respond  Acknowledgement by the primary care team of the possible separate needs of carers and the changing nature of support required.  There was no agreement between workshops about whether the new anti-dementia medication meant that early treatment had become more effective than late treatment, nor was there agreement on a suitable test.  Two workshops made explicit comments about the ethics of screening, the problems of obtaining informed consent, the threat of screening to the psychological stability of patients and the problem of overcoming taboos about dementia to allow screening to be accepted. |
| **Iracleous (2010).** Primary care physicians' attitudes towards cognitive screening: findings from a national postal survey. *Int J Geriatr Psychiatry.* 2010;25(1):23-29. | The majority of physicians ‘Agreed’ or ‘Strongly Agreed’ that cognitive impairment assessment is important in primary care (89%), and ‘Disagreed’ or ‘Strongly Disagreed’ that it should be left to specialists (92%). However, 35% were undecided when asked if assessment in primary care would lead to better outcomes. | **Screening for cognitive impairment is important in the primary care setting** Strongly disagree 2  Disagree 2  Undecided 6  Agree 49  Strongly agree 40 **There is no point/role of cognitive screening in primary care** Strongly disagree 48  Disagree 44  Undecided 5  Agree 2  Strongly agree 1 **Cognitive screening should be left to specialists** Strongly disagree 38  Disagree 54  Undecided 6  Agree 2  Strongly agree 1 **Cognitive screening in primary care leads to better outcomes** Strongly disagree 2  Disagree 3  Undecided 35  Agree 44  Strongly agree 17 | Female physicians were more likely to have a positive attitude towards cognitive assessment. | Respondents were asked to specify the ideal or optimal length of time required to administer a cognitive screening tool in the primary care setting. Based on 241 responses, the average ideal time was 9.23 min (range 1–45 min).  There is a need for better cognitive assessment tools for the primary care setting. |
| **Justiss (2009).**  Patients' attitudes of dementia screening across the Atlantic. Int J Geriatr Psychiatry. 2009;24(6):632-637. | Caregiver support dynamics for participants may have inﬂuenced responses. Although more of the UK participants were married, they indicated a signiﬁcantly higher perceived loss of independence associated with dementia screening than the US. The perceived role of the spouse as a potential caregiver may be a potential barrier to the screening process   Both groups perceived dementia screening as beneﬁcial (p¼0.218). After controlling for prior experience with dementia, acceptance and stigma were marginalized. | **Comparison of primary care patients’ domain scores between the US and the UK sites adjusting for age, gender, education plus relative or friend with AD, more problems with memory for same age** US (n=125)  Mean dementia screening acceptance score (SD) 60.6 (17.7)  Mean benefit score (SD) 69.0 (9.8)  Mean stigma score (SD) 37.5 (10.3)  Mean loss of independence score (SD) 54.0 (11.4)  Mean suffering score (SD) 55.9 (14.5)  UK (n=120)  Mean dementia screening acceptance score (SD) 66.9 (18.0)  Mean benefit score (SD) 66.5 (14.5)  Mean stigma score (SD) 41.2 (10.3) Mean loss of independence score (SD) 59.6 (11.2)  Mean suffering score (SD) 62.3 (13.4)  P-value (adjusted p-value for age, gender, education for friend with AD & more problems with memory for same age) Mean dementia screening acceptance score 0.053 Mean benefit score 0.310 Mean stigma score 0.088 Mean loss of independence score 0.004 Mean suffering score 0.001 | NA | Low acceptance rates and high rates of perceived harms might be a signiﬁcant barrier for the introduction of treatment or preventive methods for dementia in the future within the US and UK health care systems.  UK participants were more accepting of dementia screening. However, despite the higher acceptance of screening, the UK group considered a diagnosis of dementia to carry more stigma, and greater impact on independence and suffering.  Need for evidence showing that the beneﬁts for dementia screening outweigh the potential harm |
| **Krohne (2011).** Cognitive screening tests as experienced by older hospitalised patients: a qualitative study. *Scand J Caring Sci.* 2011;25(4):679-687. | The significance of the screening was not fully understood by the patient until it was over. This lack of understanding may possibly increase the threat the screening experience poses to an individual’s dignity of identity and, more specifically, to their self-respect.  Some patients had been screened previous to this hospital stay, making them able to compare their present efforts with those in an earlier experience | NA | (P1F) Yes, (I’ve done it) once before – and things might have been a little clearer for me then…And now I’m not able to write, so just writing my name is a problem. (P1F) ‘No, I wasn’t told. I don’t know’.  (P1F) ‘It is probably to do a little bit of research on what we remember, and…if our heads are where they are supposed to be…’.  (P2M) ‘[I]f I was to guess (...) it has something to do with memory?’.  (P3M) ‘I had more than enough on my plate trying to get out of there!’  (P3M): (I remember it) vaguely – I’ve really experienced so much weird whilst being here – so much out of the ordinary. What I want to say is…so I haven’t really got a clear idea about everything. (P4F): I got the impression that I passed the test. Yes. Or you could say it was examination questions, right? KK: You got sort of a school feeling? P4F: Yes. I didn’t pass on all of it – and I really want to be that person who passes…  (P4K) Yes, yes – it’s very…yes, I didn’t like it. Train, rabbit…no, let’s see it was train, rabbit. No, it was house, train…hmmm, let’s see; I think it must be house, rabbit, and train. KK: Yes, you’ve got it! P4K: Yes, I have – but it still annoys me. I have to admit.  (P4M) then I was ﬁnished! KK: You were ﬁnished? Where did you come to a stop? P4M: It was that subtraction task… KK: Is this something you’ve thought about afterwards? P4M: Some… KK: What have you been thinking? P4M: That, by Jove, I’ll get even! (P5M): I think it went well up to a certain point – and then I was ﬁnished! KK: You were ﬁnished? Where did you come to a stop? P4M: It was that subtraction task… KK: Is this something you’ve thought about afterwards? P4M: Some… KK: What have you been thinking? P4M: That, by Jove, I’ll get even!  (P5M) ‘A test ... that’s what I thought’.  (P6F) ‘like Alzheimer questions’.  (P7F) You know when you get to an age like me and you’re forgetful, it isn’t easy to start adding and subtracting and stuff’ (P9F) ‘wasn’t it just questions?’  (P10F) ‘didn’t feel like the stupidest… (person alive)’.  (P10F) ‘It’s okay to be screened ‘cause then [the occupational therapist] gets to see what I really need help to do’.  (P11M) ‘I didn’t really do well on the screening’:  (P11M): No, that’s something I just close my eyes to and forget – elegantly. KK: Right… P11M: That’s something I’ll suppress. KK: What do you mean you suppress? Stuff you don’t manage or these screenings?  (P11M): Nooo, not the screening. Stuff I can’t manage, that’s what I’ll elegantly suppress. …I’m not bothered enough to let it annoy me.  (P14M) It wasn’t that it couldn’t be done, but at my age…I got tired – this is a weariness I carry with me everywhere (and it kicks in every time) I’m exposed to something complicated. (P15F) ‘…she tested my head, that’s what she did’.  (P17M) I was perhaps a little proud because I managed to do these things faster than the given time…Even though I wasn’t much faster, I was faster. | The occupational therapists’ initial presentation is not fully understood by the older patient, leaving him or her to interpret the screening test in light of the questions answered and the tasks solved. Screening test operators should ensure that the signiﬁcance of the screening is fully understood by the patient before it is implemented. The patients found the screening strenuous. Feelings ranging from shame and irritation to pride and relief. |
| **Lawrence (2003).**  Is large-scale community memory screening feasible? Experience from a regional memory-screening day. *J Am Geriatr Soc.* 2003;51(8):1072-1078. | Most participants volunteered to be screened because they wanted to know more about their memory status and were concerned about the possibility of memory loss. | **Primary Care Physician Response to the Screen According to Participants (n=42)** Referred for more testing n=23, 54.8% Monitoring changes in memory n=8, 19.0% Diagnosis perceived as not relevant to participant n=7, 16.7% Other n=4, 9.5%  More Hispanic Americans (85% [29of33]) came to the screening purposefully rather than by chance when compared to African American (58% [15/26], Afro-Caribbean 58% [18 of 31] and European American participants 46% [13 of 29]; (w2[6, N =119] =13.69, P =.03).  Eighty-nine percent (n=105) of the participants stated that they valued the screening and 92% (n =109) would recommend screening to others. Only 4% (n =5) indicated that they did not value screening at all. All who disagreed (n =3) that others should be screened were European Americans.  Most participants (65%,n =77) agreed to be screened because they or family members were concerned about participants’ memory. Other participants (29%,n=34) simply decided to take advantage of the opportunity to be screened. The remainder (6%,n =7) of the sample provided no clear reason for participating in screening. | ‘‘So many people are confused. My sister is very depressed with memory impairment.’’  ‘‘there are so many cases of Alzheimer’s disease these days.’’  ‘‘I don’t want to forget my children’’  ‘‘I’m over 50 with no children. I need to know how to be prepared.’’  ‘‘I have had concerns about my memory ... Both my parents have Alzheimer’s disease.’’  ‘‘My wife wanted me to come because I forget things sometimes.’’  ‘‘catch it before it’s too late.’’  ‘‘I think I have it (AD).’’  ‘‘It is dangerous, especially driving with memory problems.’’ “It got us started, it was very painless, not much paperwork like at the hospital”.  Most of the participants (82%, 97 of 119) were certain about their course of action if the screening results were positive. Their responses were ‘‘find out more,’’ seek professional help,’’ ‘‘see a doctor or specialist,’’ ‘‘get medications,’’ ‘‘do whatever is necessary,’’ ‘‘talk to my family,’’ ‘‘try to correct it,’’ ‘‘use complementary treatments,’’ or ‘‘do nothing, nothing can be done”. | Most participants volunteered to be screened because they wanted to know more about their memory status and were concerned about the possibility of memory loss.   Some of those scoring high or retest appeared surprised by the result, denying that they had significant memory difficulties, and although not overtly anxious or angry, they were reluctant to follow through with further evaluation. Even those who were initially willing to seek follow-up were likely to forget to do so.  The community setting and voluntary nature of the screening may have selected healthier individuals who were sufficient capable to attend the event  the screening program was costly in terms of volunteer staff time, the cost of development of the program, and publicity, but this has to be weighed against potential cost savings of earlier treatment.  Limitation to the effectiveness of a large-scale screening project derives from the poor follow-up of screening recommendations. |
| **Manthorpe (2003).** The implications of the early recognition of dementia for multiprofessional teamworking: conflicts and contradictions in practitioner perspectives. *Dementia.* 2003;2(2):163-180. | Opportunistic identiﬁcation was also seen as a rationing device Routine checks would be a ‘huge drain’ if all PHCTs were to undertake them, and whole population screening would not work. Who to screen was very much linked to cost efﬁciency.   Practitioners needed to build up expertise, the primary care team needed training and professionals needed to be supervised.  Screening as an activity that should be non-traumatic, non-invasive, acceptable and simple. | **Identification of professional group appropriate to undertake screening. Screening responsibility and consensus on role – frequency of times mentioned** Practice nurse n=10 Health visitor n=6 Community/district nurse n=5 Community psychiatric nurse (including dementia nurse) n=5 General practitioner n=2 Other (unspecified) nurses n= 2 Social worker n=1 Memory clinic n=1 Lay person/voluntary organization n=2 | ‘to diagnose it, you must think of it’.  ‘not keen on a formal programme . . . [we] already cannot cope with the 75 and over checks therefore education is a priority’.  the aim should be to make the whole Primary Health Care Team (PHCT) ‘aware of dementia and then to look out for it’.  Flexibility (is) important: must pick up on cues from carers, pharmacists and receptionists’.  ‘if the family requested it’  ‘awareness of what other people’s responsibilities are’ | Some workshops saw opportunism as particularly appropriate for deﬁned age groups (for example, those aged 75 or more) or on request.   Number of difﬁculties with screening related to its operation in practice, whether it is an effective use of resources or beneﬁts individual patients. Screening raised questions about team working and roles.  The practice nurse was identiﬁed as the practitioner most appropriate to take on screening for dementia.  Many groups thought it appropriate to refer individuals to social services, erroneously seeing them as sources of help and expertise on ﬁnancial matters. |
| **Martinez-Lage (2010).**  Assessing physician attitudes and perceptions of Alzheimer's disease across Europe. *J Nutr Health Aging.* 2010;14(7):537-544. | A slight majority (56%) of physicians indicated that they felt that routine screening for people aged >65 years is extremely important or very important, and 42% of physicians agreed that screening should be routinely performed on every person when they reach the age of 65 years. | I. I would want to know as early as possible if a family member had AD France GP 78  France Specialist 90  Germany GP74  Germany Specialist 78  Italy GP 84 Italy Specialist 80  Spain GP 80  Spain Specialist 78  UK GP 62  UK Specialist 72  II. I would like to know as early as possible if I had AD  France GP 66  France Specialist 64  Germany GP 60  Germany Specialist 58  Italy GP 76  Italy Specialist 66  Spain GP 70  Spain Specialist 58  UK GP 48  UK Specialist 70  III. I would think there are more disadvantages than advantages to finding out if someone has AD as early as possible France GP 26  France Specialist 18  Germany GP 16  Germany Specialist 20  Italy GP 74  Italy Specialist 72  Spain GP 18  Spain Specialist 32  UK GP 44  UK Specialist 24  IV. Most people wouldn’t know the difference between the early stages of AD and normal ageing France GP 863  France Specialist 78  Germany GP 88 Germany Specialist 78  Italy GP 82  Italy Specialist 86  Spain GP 68  Spain Specialist 76  UK GP 66  UK Specialist 66  V. Most people would not recognise the early signs of AD France GP 82  France Specialist 80  Germany GP 80  Germany Specialist 78  Italy GP 74 Italy Specialist 62  Spain GP 70  Spain Specialist 52  UK GP 62  UK Specialist 64  VI. General practitioners and hospital physicians have difficulty detecting AD in its early stages France GP 58  France Specialist 62  Germany GP 52  Germany Specialist 62  Italy GP 46  Italy Specialist 62  Spain GP 56  Spain Specialist 52  UK GP 70 UK Specialist 68  VII. Specialists such as neurologists or geriatricians have difficulty detecting AD in its early stages France GP 32  France Specialist 16  Germany GP 32  Germany Specialist 24  Italy GP 32  Italy Specialist 16  Spain GP 36  Spain Specialist 34  UK GP 56  UK Specialist 26  VIII. Fear about AD keeps people from speaking with their doctor about it France GP 56  France Specialist 56  Germany GP 66  Germany Specialist 56  Italy GP 28  Italy Specialist 30  Spain GP 40  Spain Specialist 16  UK GP 76  UK Specialist 60  IX. Being diagnosed with dementia is not as stressful as being diagnosed with AD  France GP 16  France Specialist 28 Germany GP 30  Germany Specialist 32 Italy GP 42 Italy Specialist 46 Spain GP 38 Spain Specialist 22  UK GP 24  UK Specialist 10 | Results showed that a sizable majority of physicians throughout Europe, specialists and generalists alike, agree that:  1) AD is underdiagnosed and undertreated 2) patients and families are not prepared to recognise the early symptoms of the disease  3) early treatment can help to slow the progression of the disease 4) more effective treatments are needed. | In the United Kingdom not a single physician agreed that AD is over treated.  68% of physicians in the United Kingdom agreed that the government does not invest nearly enough in treatment  50% of physicians in the United Kingdom felt that their government is a barrier to those seeking AD medications  High percentage of physicians believe that most people struggle to recognise the early signs of AD and find it difficult to differentiate these symptoms from signs of normal ageing  There is substantial opposition to the implementation of routine screening programmes for AD |
| **Welkenhuysen (1997).**  Attitudes toward predictive testing for Alzheimer's disease in a student population. . *Psychiatr Genet.* 1997;7(3):121-126. | 51.8% of the students thought that the development of a predictive test for AD was rather or totally unimportant; 48.2% considered the development of such a test as rather or very important. | 27.2% of responses dealt with the expectation that the knowledge of being a carrier for AD is too stressful and depressing.  About one quarter of the explanations given by the students (25.8%) can be considered as arguments in favour. Most frequently, these arguments dealt with the ability to make plans for the future and to prepare oneself for the disease (15.9% explanations).  More than 40% of the sample was situated on the negative side of the scale, indicating that the disadvantages were, to some extent, bigger than the advantages.  The relationship between the judgement of (dis)advantages and the importance attributed to the development of a predictive test for AD, is significant (Pearson; r = 0.72, p < 0.001).  Relationship between personal intention and the two other attitude variables is significant: a more positive personal intention corresponds with a higher degree of importance attributed to the development of a predictive test for AD (Pearson; r=0.64, p < 0.001) and with a more positive judgement of the advantages of such a test (Pearson; r=0.62, p < 0.001).  Correlational analyses (Pearson) showed that the qualitative evaluation was positively related with the quantitative estimation of their own chance (r = 0.51; p < 0.001). These two measures of risk perception are not related to sociodemographic variables, nor being related to an AD patient, nor the total score for knowledge on the ADK test.  Stepwise regression analyses have shown that the total score for knowledge about AD on the ADK test is the best predictor for all three attitudinal variables. | Less than one fifth of the remaining sample (18.8%) thought between 20 and 30 years the most appropriate age to have a test for AD, 25.0% considered between 30 and 40 years as the most appropriate age, between 40 and 50 years was chosen by 19.6% and, according to 36.6%, the best age is between 50 and 60 years. each student's own chance of developing AD, the following results were obtained: 1.8% of the total sample thought they had no chance at all, 28.2% thought of the chance as very small 41.1% considered the chance as small, 27% thought the chance moderate and 1.8% considered it very big. | The low level of knowledge and the divergence in attitudes and opinions reflect the complexity and the problems. |
| **Williams (2010).** Willingness to be screened and tested for cognitive impairment: cross-cultural comparison. *Am J Alzheimers Dis Other Demen.* 2010;25(2):160-166. | Most of our participants (65%,n =77) came to the screening because of concern about their memory rather than as a routine health check. | More Hispanic Americans (85% [29of33]) came to the screening purposefully rather than by chance when compared to African American (58% [15/26], Afro-Caribbean 58% [18 of 31] and European American participants 46% [13 of 29]; (w2[6, N =119] =13.69, P =.03).  Eighty-nine percent (n=105) of the participants stated that they valued the screening and 92% (n=109) would recommend screening to others.  4% (n=5) indicated that they did not value screening at all.  Most participants (65%,n =77) agreed to be screened because they or family members were concerned about participants’ memory.  Other participants (29%,n=34) simply decided to take advantage of the opportunity to be screened  6% (n=7) of the sample provided no clear reason for participating in screening.  39% (46 of 119) replied that they would seek professional help following positive screening.  Respondents said they would not seek help (7%, 8 of 119), did not know what they would do (5%, 6 of 119), or planned to delay seeking help and preferred to use the information as a baseline for future evaluations or just for their own knowledge (2%, 2 of 119).  Most of the participants (82%, 97 of 119) were certain about their course of action if the screening results were positive. (6%, 7 of 119) preferred complementary approaches rather than professional help | ‘‘So many people are confused. My sister is very depressed with memory impairment.’’  Participants were willing to be screened to ‘‘take care of’’ themselves, ‘‘detect early problems,’’ or because they or others were concerned about their memory.  ‘‘I don’t want to forget my children’’  ‘‘I’m over 50 with no children. I need to know how to be prepared.’’  ‘‘catch it before it’s too late.’’  ‘‘I would get vitamins or food to help the memory,’’   ‘‘I would try the nutritionist first,’’   ‘‘I don’t think today’s medications do anything. They’re not advanced. Maybe some herbs,’’   ‘‘Drink more Mon Avie’ | If perceived susceptibility is important in explaining willingness to be screened and evaluated, then resources would be allocated to educating the public about AD  About half of all adults studied regardless of ethnic group membership, cognitive screening and follow-up were declined  Most participants were screened because of concern about their memory rather than as a routine health check. |
