# Supplementary material for: Attitudes and preferences towards screening for dementia: a systematic review of the literature
Source: BMC Geriatr. 2015 Jun 16;15:66. doi: 10.1186/s12877-015-0064-6 (PMC4469007; doi:10.1186/s12877-015-0064-6)
Supplement: Additional file 2: — PRISMA checkist. [file 12877_2015_64_MOESM2_ESM.docx]

**Appendix 2: Search strategy Medline Search – Patients & Carer Perceptions**

|  | exp DEMENTIA/ |
| --- | --- |
|  | exp ALZHEIMER DISEASE/ |
|  | dement*.ti,ab |
|  | alzheimer*.ti,ab |
|  | (Cognit* AND (disord* OR impair* OR declin* OR function*)).ti,ab |
|  | 1 OR 2 OR 3 OR 4 OR 5 |
|  | exp NEUROPSYCHOLOGICAL TESTS/ |
|  | (Neuropsych* adj3 (Test* OR assess*)).ti,ab |
|  | (psychologic* adj3 (test* OR assess*)).ti,ab |
|  | exp PSYCHOLOGICAL TESTS/ |
|  | exp GERIATRIC ASSESSMENT/ |
|  | "geriatric assess*".ti,ab |
|  | exp QUESTIONNAIRES/ |
|  | questionnaire*.ti,ab |
|  | exp SELF-ASSESSMENT/ |
|  | exp SELF-EXAMINATION/ |
|  | (self adj3 assess*).ti,ab |
|  | (self adj3 exam*).ti,ab |
|  | exp INTERVIEWS AS TOPIC/ |
|  | ((telephone interview*)).ti,ab |
|  | 7 OR 8 OR 9 OR 10 OR 11 OR 12 OR 13 OR 14 OR 15 OR 16 OR 17 OR 18 OR 19 OR 20 |
|  | exp MASS SCREENING/ |
|  | ((mass screen*)).ti,ab |
|  | "population screen*".ti,ab |
|  | screen*.ti,ab |
|  | detect*.ti,ab |
|  | 22 OR 23 OR 24 OR 25 OR 26 |
|  | "3 word recall".ti,ab |
|  | "word recall".ti,ab |
|  | "7-minute screen".ti,ab |
|  | "7MS".ti,ab |
|  | "6 item cognitive impairment test".ti,ab |
|  | "6 CIT".ti,ab |
|  | "AB cognitive screen".ti,ab |
|  | "abbreviated mental test".ti,ab |
|  | "ADAS-cog".ti,ab |
|  | "AD8 informant interview".ti,ab |
|  | "animal fluency test".ti,ab |
|  | "brief alzheimer* screen".ti,ab |
|  | "brief cognitive scale".ti,ab |
|  | "clinical dementia rating scale".ti,ab |
|  | "clinical dementia test".ti,ab |
|  | "community screening interview for dementia".ti,ab |
|  | "cognitive abilities screening instrument".ti,ab |
|  | "cognitive assessment screening test".ti,ab |
|  | "cognitive capacity screening examination".ti,ab |
|  | "clock drawing test".ti,ab |
|  | "deterioration cognitive observee".ti,ab |
|  | "Dem Tect".ti,ab |
|  | "fuld object memory evaluation".ti,ab |
|  | "general practitioner assessment of cognition".ti,ab |
|  | "GPCOG".ti,ab |
|  | "Hopkins verbal learning test".ti,ab |
|  | "HVLT".ti,ab |
|  | "IQCODE".ti,ab |
|  | "mattis dementia rating scale".ti,ab |
|  | "memory impairment screen".ti,ab |
|  | "minnesota cognitive acuity screen".ti,ab |
|  | "mini-cog".ti,ab |
|  | "mini-mental state exam*".ti,ab |
|  | "mmse".ti,ab |
|  | "modified mini-mental state exam".ti,ab |
|  | "3MS".ti,ab |
|  | "neurobehavioural cognitive status exam*".ti,ab |
|  | "cognistat".ti,ab |
|  | "quick cognitive screening test".ti,ab |
|  | "QCST".ti,ab |
|  | "rapid dementia screening test".ti,ab |
|  | "RDST".ti,ab |
|  | "repeatable battery for the assessment of neuropsychological status".ti,ab |
|  | "RBANS".ti,ab |
|  | "rowland universal dementia assessment scale".ti,ab |
|  | "rudas".ti,ab |
|  | "self-administered gerocognitive exam*".ti,ab |
|  | ("self-administered" AND "SAGE").ti,ab |
|  | "self-administered computerized screening test for dementia".ti,ab |
|  | "short and sweet screening instrument".ti,ab |
|  | "sassi".ti,ab |
|  | "short cognitive performance test".ti,ab |
|  | "syndrome kurztest".ti,ab |
|  | "six item screener".ti,ab |
|  | "short memory questionnaire".ti,ab |
|  | ("short memory questionnaire" AND "SMQ").ti,ab |
|  | "short orientation memory concentration test".ti,ab |
|  | "s-omc".ti,ab |
|  | "short blessed test".ti,ab |
|  | "short portable mental status questionnaire".ti,ab |
|  | "spmsq".ti,ab |
|  | "short test of mental status".ti,ab |
|  | "telephone interview of cognitive status modified".ti,ab |
|  | "tics-m".ti,ab |
|  | "trail making test".ti,ab |
|  | "verbal fluency categories".ti,ab |
|  | "WORLD test".ti,ab |
|  | "time and change test".ti,ab |
|  | "modified world test".ti,ab |
|  | "symptoms of dementia screener".ti,ab |
|  | "dementia questionnaire".ti,ab |
|  | /or 28 - 98 |
|  | exp DIAGNOSTIC TESTS, ROUTINE/ |
|  | exp DIAGNOSIS/ OR exp EARLY DIAGNOSIS/ |
|  | diagnos*.ti,ab |
|  | (routine adj3 diagnos*).ti,ab |
|  | /or 7 - 103 |
|  | exp PATIENTS/ |
|  | patient*.ti,ab |
|  | inpatient*.ti,ab |
|  | outpatient*.ti,ab |
|  | client*.ti,ab |
|  | user*.ti,ab |
|  | consumer*.ti,ab |
|  | exp PERSONS/ |
|  | person*.ti,ab |
|  | personal*.ti,ab |
|  | people*.ti,ab |
|  | individual*.ti,ab |
|  | exp CAREGIVERS/ |
|  | caregiver*.ti,ab |
|  | (care ADJ giver*).ti,ab |
|  | carer*.ti,ab |
|  | guardian*.ti,ab |
|  | exp FAMILY/ |
|  | family*.ti,ab |
|  | relative*.ti,ab |
|  | relation*.ti,ab |
|  | spouse*.ti,ab |
|  | partner*.ti,ab |
|  | husband*.ti,ab |
|  | wife*.ti,ab |
|  | child*.ti,ab |
|  | daughter*.ti,ab |
|  | son*.ti,ab |
|  | father*.ti,ab |
|  | mother*.ti,ab |
|  | brother*.ti,ab |
|  | sister*.ti,ab |
|  | sibling*.ti,ab |
|  | "next of kin".ti,ab |
|  | friend*.ti,ab |
|  | /or 105 - 139 |
|  | view*.ti,ab |
|  | experience*.ti,ab |
|  | perspective*.ti,ab |
|  | exp PERCEPTION/ OR exp SOCIAL PERCEPTION/ |
|  | perception*.ti,ab |
|  | satisfaction*.ti,ab |
|  | perceived.ti,ab |
|  | concern*.ti,ab |
|  | issue*.ti,ab |
|  | exp ATTITUDE/ |
|  | attitude*.ti,ab |
|  | perceiv*.ti,ab |
|  | belief*.ti,ab |
|  | aware*.ti,ab |
|  | understand*.ti,ab |
|  | concept*.ti,ab |
|  | knowledge*.ti,ab |
|  | 141 OR 142 OR 143 OR 144 OR 145 OR 146 OR 147 OR 148 OR 149 OR 150 OR 151 OR 152 OR 153 OR 154 OR 155 OR 156 OR 157 |
|  | exp ATTITUDE TO HEALTH/ OR exp PATIENT SATISFACTION/ OR exp PHYSICIAN-PATIENT RELATIONS/ |
|  | exp "PATIENT ACCEPTANCE OF HEALTH CARE"/ |
|  | exp PUBLIC OPINION/ |
|  | exp PERSONAL SATISFACTION/ |
|  | exp PATIENT SATISFACTION/ |
|  | exp PROFESSIONAL-PATIENT RELATIONS/ OR exp PROFESSIONAL-FAMILY RELATIONS/ |
|  | exp CONSUMER PARTICIPATION/ OR exp CONSUMER SATISFACTION/ |
|  | exp PATIENT EDUCATION AS TOPIC/ OR exp PATIENT COMPLIANCE/ |
|  | (patient AND information).ti,ab |
|  | ((patient* OR consumer* OR client) AND (compliance* OR participat* OR accept* OR refus*)).ti,ab |
|  | 159 OR 160 OR 161 OR 162 OR 163 OR 164 OR 165 OR 166 OR 167 OR 168 |
|  | 140 AND 158 |
|  | 169 OR 170 |
|  | 6 AND 104 AND 171 |
|  | 6 AND 104 AND 170 |
|  | 6 AND 104 AND 158 |
|  | exp QUALITATIVE RESEARCH/ |
|  | exp INTERVIEWS AS TOPIC/ OR exp FOCUS GROUPS/ |
|  | qualitative*.ti,ab |
|  | exp DATA COLLECTION/ |
|  | survey*.ti,ab |
|  | exp QUESTIONNAIRES/ |
|  | ethnographic.ti,ab |
|  | exp OBSERVATION/ |
|  | observation*.ti,ab |
|  | (grounded adj3 theory).ti,ab |
|  | "life experience".ti,ab |
|  | phenomenologic*.ti,ab |
|  | narrative*.ti,ab |
|  | discourse*.ti,ab |
|  | story*.ti,ab |
|  | stories*.ti,ab |
|  | 175 OR 176 OR 177 OR 178 OR 179 OR 180 OR 181 OR 182 OR 183 OR 184 OR 185 OR 186 OR 187 OR 188 OR 189 OR 190 |
|  | 172 AND 191 |
|  | 173 AND 191 |
|  | 6 AND 27 AND 171 AND 191 |
|  | 105 OR 106 OR 107 OR 108 OR 109 OR 110 OR 111 OR 114 OR 117 OR 118 OR 119 OR 120 OR 121 OR 122 OR 123 OR 125 OR 127 |
|  | 6 AND 27 AND 158 AND 191 AND 195 |
|  | 27 OR 100 OR 101 OR 102 OR 103 |
|  | 6 AND 171 AND 191 AND 197 |
|  | 6 AND 197 AND 158 AND 191 AND 195 |
