# Supplementary material for: Attitudes and preferences towards screening for dementia: a systematic review of the literature
Source: BMC Geriatr. 2015 Jun 16;15:66. doi: 10.1186/s12877-015-0064-6 (PMC4469007; doi:10.1186/s12877-015-0064-6)
Supplement: Additional file 4 — Search strategy Medline Search – Patients & Carer Perceptions. [file 12877_2015_64_MOESM4_ESM.docx]

## Appendix 4: List of funders for primary studies

| **First Author (Year)** | **Funding sponsor** |
| --- | --- |
| Boise (1999) | Supported by a grant from the National Alzheimer's Association (Grant PRG-95-186), and the Oregon Alzheimer Disease Center (NIA Grant #P30 AG08017). |
| Boise (2010) | Supported by grant no. R03 HS016007-01 from the Agency for Health Care Research and Quality; grant no. P30 AGO080 (Oregon Alzheimer’s Disease Center) from National Institute on Aging; the Oregon Clinical and Translational Research Institute; and grant no. UL1 RR024140 from the National Center for Research Resources, a component of the National Institutes of Health and National Institutes of Health Roadmap for Medical Research. |
| Bond (2010) | The IMPACT study was funded by Pfizer Inc and Eisai Inc. The article was funded by Pfizer Inc and Eisai Inc. Editorial support was provided by Tom Claus, PhD, of PAREXEL and was funded by Pfizer Inc and Eisai Inc. |
| Borson (2007) | This study was funded by an investigator initiated grant from Ortho McNeil (GAL-ALZ-419). |
| Boustani (2008) | This study was supported by grants (P30AG024967; R01AG029884-01) from the National Institute on Aging, the Program on Aging at the University of North Carolina at Chapel Hill and the Foundation of Hope. Dr Boustani was supported by the Paul B. Beeson K23 Career Development Award # 1-K23- AG026770-01. Mr Hopkins was supported by the American Federation for Aging Research, Medical Student Summer Research Training in Aging Scholarship. |
| Boustani (2003) | American Federation for Aging Research |
| Boustani (2011) | Supported by National Institute on Aging (NIA) Grant R01AG029884-01. Dr. Austrom was supported in part by National Institutes of Health Grants P30 AG10133 and P30AG024967 from the NIA to the IU Roybal Center. |
| Brodaty (1994) | RADGAC grant from the commonwealth Department of health, Housing, Local Government and Community Services under the auspices of the Alzheimer's Association (Australia) |
| Bush (1997) | This research was funded by the National Health Research and Development Program, Project No. 6606-5471-55. |
| Cahill (2008) | Not specified |
| Carpenter (2011) | Dr Carpenter was supported by the Washington University Goldfarb Patient Safety Award. |
| Dale (2008) | The John A. Hartford Center of Excellence in Geriatric Medicine Pilot Grant No.2003-0201, 27239 (Dale) |
| Dale (2006) | William Dale: The John A. Hartford Center of Excellence in Geriatric Medicine Pilot Grant #2003–0201, 27239; National Institutes of Health/National Institute on Aging (NIA) Paul B. Beeson Career Development Awards in Aging, 1 K23 AG 024812–01. Emily K. Hill: Summer Training on Aging Research Topics Mental Health Program (START-MH), 2004. Greg A. Sachs: Multiple funding sources from NIA, Agency for Healthcare Research and Quality, The John A. Hartford Foundation, and the Donald W. Reynolds Foundation. |
| Downs (2000) | Allied Dunbar for funding the training initiative at the University of Stirling. |
| Fowler (2012) | This work was supported by a Paul A. Beeson Career Development Award in Aging (K23AG26770–01) from the National Institute on Aging (NIA), the Hartford Foundation, and the Atlantic Philanthropy. |
| Galvin (2008) | The Alzheimer Association (IIRG 03-5578). Additional support was provided by the National Institute on Aging (AG20764, AG03991, and AG05681), the American Federation for Aging Research, and by a generous gift from the Alan A and Edith L Wolff Charitable Trust. |
| Galvin (2012) | This work was supported by grants from the National Institute on Aging at the National Institutes of Health (P01 AG03991, P01 AG026276, and P50 AG05681). |
| Hansen (2008) | Not specified |
| Holsinger (2011) | Not specified |
| Iliffe (1994) | Not specified |
| Iliffe (2003) | This project was funded and organized by Exerpta Medica, using an educational grant from Pfizer Ltd. |
| Iracleous (2010) | Funding for this project was obtained from the Department of Family and Community Medicine at Sunnybrook Health Sciences Centre/University of Toronto. |
| Justiss (2009) | Supported by grants from the National Institute on Aging; (Roybal Grant, P30AG024967) and 1R01AG029884-01. Dr Boustani was supported by the Paul B. Beeson K23 Career Development Award # 1-K23-AG026770-01. |
| Krohne (2011) | This research was supported by a grant from The Research Council of Norway (176515). |
| Lawrence (2003) | Not specified |
| Manthorpe (2003) | The workshops were funded by an educational grant from Pfizer and Eisai. |
| Martinez-Lage (2010) | The IMPACT study was sponsored by Pfizer Inc and Eisai Inc. This article was funded by Pfizer Inc and Eisai Inc. Editorial support was provided by Bill Kadish, MD, of PAREXEL and was funded by Pfizer Inc and Eisai Inc. |
| Williams (2010) | Supported by funds from the Johnnie B. Byrd Alzheimer Disease Center and Research Institute, Phase I, entitled Memory Screening Studies to Detect Progression of Mild Cognitive Impairment in Patients (R. Tappen, P.I.). |
| Welkenhuysen (1997) | Not specified |
