# Supplementary material for: Attitudes and preferences towards screening for dementia: a systematic review of the literature
Source: BMC Geriatr. 2015 Jun 16;15:66. doi: 10.1186/s12877-015-0064-6 (PMC4469007; doi:10.1186/s12877-015-0064-6)
Supplement: Additional file 6 — Data extraction for included studies. [file 12877_2015_64_MOESM6_ESM.docx]

## **Appendix 6: Systematic review protocol**

## Steven Martin, Sarah Kelly, Ayesha Khan, Sarah Cullum, Tom Dening, Greta Rait, Chris Fox, Cornelius Katona, Theodore Cosco, Carol Brayne, and Louise Lafortune. *Attitudes and Preferences towards screening for dementia: a systematic review of the literature*

## **Review question(s)**

Examine the attitudes and preferences of the general public, health care professionals, people with dementia and their carers towards population screening for dementia

# Searches

## The search strategies had no time or language limits, covered all types of dementia. A comprehensive international literature review using a systematic review methodology was undertaken. Fifteen electronic bibliographic databases were searched (MEDLINE, EMBASE, CINAHL, CDR, Cochrane and Campbell Collaboration databases; PsycINFO, Social Sciences Citation Index, Web of Science, Bibliomap, DoPHER, TRoPHI); hand searches of key journals were also undertaken. Reference lists of all included studies were scanned for relevant articles and reports.

# Condition or domain being studied

## We are reviewing evidence pertaining to population screening, i.e. testing of asymptomatic individuals, for any sub types of dementia.

# Participants/population

## Inclusion: Health and social care professionals, members of the general public, people with dementia, carers or formal carers were included irrespective of their age, education, cognitive or dementia diagnostic status, or the setting in which the study was conducted. In addition, studies that assess health professionals’ perceptions, views and experiences were also included.

## Exclusion: There are no populations excluded from this review

# Intervention(s), exposure(s)

## This review only includes pen and pencil tests or any tests that can currently be easily administered in primary or community care settings to screen for dementia. Genetic tests and biomarkers are excluded.

# Comparator(s)/control

## Any comparator or no comparator – we are interested in attitudes and preferences towards population screening in any context, irrespective of whether the study includes a control/comparator arm or scenario

# Types of study to be included initially

## All study designs (qualitative, quantitative randomized experimental, quantitative non-randomized controlled, quantitative observation, and mixed methods) were eligible for inclusion. Only published sources were considered. Opinion-based papers were excluded.

# Context

## This review includes any test that can currently be easily administered in primary or community care settings to screen for dementia.

# Primary outcome(s)

## Perceptions, views and/or attitudes and/or experiences of patients and carers, members of the general public, and health and social care professionals were analysed with particular attention given to 1) their experience of screening (receiving or administering), 2) their view of population screening as an intervention (positive, negative), 3) quotes in support of views and perspectives, and 4) outcomes (views considered by authors as being associated with positive or false negative results).

# Secondary outcomes

## Ethical, moral and cultural issues; practical implications in terms of knowledge, organisation of health and social care (e.g. accessibility of diagnosis services, information and support; etc.), resources and funding in the context of the perception of patients, members of the general public, and carers and practitioners.

# Data extraction, (selection and coding)

## All the citations identified in the search were downloaded into EndNote and screened for inclusion by two reviewers, who worked independently. All titles and abstracts were screened for inclusion by SM and LL. The full text of articles identified as either relevant or possibly relevant from the title and abstract were obtained and assessed to determine whether it met the inclusion criteria. Discrepancies between the authors were resolved via discussion at both stages.

# Risk of bias (quality) assessment

## Two reviewers independently assessed study quality using a checklist adapted by Bunn et al. (2012), building on the Spencer et al. (2003) framework for assessing quality; the overall reliability and usefulness of the study to the research questions was graded as low, medium or high. We included all studies regardless of their quality. As a broad range of study designs have been used in this area of healthcare, the use of a single checklist, in contrast to individual checklists for each study design, was considered more appropriate.

# Strategy for data synthesis

## A narrative synthesis of data from all included studies was undertaken by SM. This was done to provide a detailed summary and comparison of attitudes and preferences across studies. We then analysed the findings and discussion sections of the papers by identifying key themes represented in data (i.e. quotations) and the statements made within the discussion. We did not adopt a line-by-line analysis in which codes are assigned to each line of text as we did not feel that such intense scrutiny of content would have enhanced our collection of descriptive and interpretive data.

# Analysis of subgroups or subsets

## Data were divided into a) those assessing the views of clinicians and healthcare professionals, and b) those assessing the views of patients, carers and the general public.

# Funding sources/sponsors

# This article presents independent research funding by the Alzheimer’s Society (Project grant 129) and supported by the National Institute for Health Research Collaborations for Leadership in Applied Health Research and Care (CLAHRC) for Cambridgeshire and Peterborough.

# Conflicts of interest

# The review team declares no conflicts of interest.

# Bibliography

## Bunn et al., (2012) *Psychosocial Factors That Shape Patient and Carer Experiences of Dementia Diagnosis and Treatment: A Systematic Review of Qualitative Studies. PLoS Med 9(10): e1001331. doi:10.1371/journal.pmed.1001331*

## Spencer L, Ritchie J, Lewis J, Dillon L, eds. Quality in qualitative evaluation: a framework for assessing research evidence. London: UK Government Chief Social Researcher’s Office; 2003.
