# Supplementary material for: Attitudes and preferences towards screening for dementia: a systematic review of the literature
Source: BMC Geriatr. 2015 Jun 16;15:66. doi: 10.1186/s12877-015-0064-6 (PMC4469007; doi:10.1186/s12877-015-0064-6)
Supplement: Additional file 7: — List of funders for primary studies. [file 12877_2015_64_MOESM7_ESM.docx]

**Box 1. Wilson and Jungner classic screening criteria^10^**

1. The condition sought should be an important health problem.
2. There should be an accepted treatment for patients with recognized disease.
3. Facilities for diagnosis and treatment should be available.
4. There should be a recognizable latent or early symptomatic stage.
5. There should be a suitable test or examination.
6. The test should be acceptable to the population.
7. The natural history of the condition, including development from latent to declared disease, should be adequately understood.
8. There should be an agreed policy on whom to treat as patients.
9. The cost of case-finding (including diagnosis and treatment of patients diagnosed) should be economically balanced in relation to possible expenditure on medical care as a whole.
10. Case-finding should be a continuing process and not a “once and for all” project.
